# Clinical impact of anti-inflammatory microglia and macrophage phenotypes at glioblastoma margins

**DOI:** 10.1093/braincomms/fcad176

**Published:** 2023-06-02

**Authors:** Imran Noorani, Kastytis Sidlauskas, Sean Pellow, Reece Savage, Jeannette L Norman, David S Chatelet, Mark Fabian, Paul Grundy, Jeng Ching, James A R Nicoll, Delphine Boche

**Affiliations:** Department of Neuromuscular Diseases, The Francis Crick Institute and University College London, London NW1 1AT, UK; Department of Neurosurgery, National Hospital for Neurology and Neurosurgery, London SO16 6AQ, UK; Clinical Neurosciences, Clinical and Experimental Sciences, Faculty of Medicine, University of Southampton, Southampton, UK; Centre for Tumour Biology, Barts Cancer Institute, Queen Mary University of London, London, UK; Clinical Neurosciences, Clinical and Experimental Sciences, Faculty of Medicine, University of Southampton, Southampton, UK; Clinical Neurosciences, Clinical and Experimental Sciences, Faculty of Medicine, University of Southampton, Southampton, UK; Histochemistry Research Unit, Clinical and Experimental Sciences, Faculty of Medicine, University of Southampton, Southampton, UK; Biomedical Imaging Unit, Southampton General Hospital, University of Southampton, Southampton, UK; Department of Cellular Pathology, University Hospital Southampton NHS Foundation Trust, Southampton, UK; Department of Neurosurgery, Wessex Neurological Centre, University Hospital Southampton NHS Foundation Trust, Southampton, UK; Department of Neurosurgery, Wessex Neurological Centre, University Hospital Southampton NHS Foundation Trust, Southampton, UK; Clinical Neurosciences, Clinical and Experimental Sciences, Faculty of Medicine, University of Southampton, Southampton, UK; Department of Cellular Pathology, University Hospital Southampton NHS Foundation Trust, Southampton, UK; Clinical Neurosciences, Clinical and Experimental Sciences, Faculty of Medicine, University of Southampton, Southampton, UK

**Keywords:** glioblastoma, macrophage, microglia, immunotherapy, intratumoural heterogeneity

## Abstract

Glioblastoma is a devastating brain cancer for which effective treatments are required. Tumour-associated microglia and macrophages promote glioblastoma growth in an immune-suppressed microenvironment. Most recurrences occur at the invasive margin of the surrounding brain, yet the relationships between microglia/macrophage phenotypes, T cells and programmed death-ligand 1 (an immune checkpoint) across human glioblastoma regions are understudied. In this study, we performed a quantitative immunohistochemical analysis of 15 markers of microglia/macrophage phenotypes (including anti-inflammatory markers triggering receptor expressed on myeloid cells 2 and CD163, and the low-affinity-activating receptor CD32a), T cells, natural killer cells and programmed death-ligand 1, in 59 human *IDH1*-wild-type glioblastoma multi-regional samples (*n* = 177; 1 sample at tumour core, 2 samples at the margins: the infiltrating zone and leading edge). Assessment was made for the prognostic value of markers; the results were validated in an independent cohort. Microglia/macrophage motility and activation (Iba1, CD68), programmed death-ligand 1 and CD4^+^ T cells were reduced, and homeostatic microglia (P2RY12) were increased in the invasive margins compared with the tumour core. There were significant positive correlations between microglia/macrophage markers CD68 (phagocytic)/triggering receptor expressed on myeloid cells 2 (anti-inflammatory) and CD8^+^ T cells in the invasive margins but not in the tumour core (*P* < 0.01). Programmed death-ligand 1 expression was associated with microglia/macrophage markers (including anti-inflammatory) CD68, CD163, CD32a and triggering receptor expressed on myeloid cells 2, only in the leading edge of glioblastomas (*P* < 0.01). Similarly, there was a positive correlation between programmed death-ligand 1 expression and CD8^+^ T-cell infiltration in the leading edge (*P* < 0.001). There was no relationship between CD64 (a receptor for autoreactive T-cell responses) and CD8^+^/CD4^+^ T cells, or between the microglia/macrophage antigen presentation marker HLA-DR and microglial motility (Iba1) in the tumour margins. Natural killer cell infiltration (CD335^+^) correlated with CD8^+^ T cells and with CD68/CD163/triggering receptor expressed on myeloid cells 2 anti-inflammatory microglia/macrophages at the leading edge. In an independent large glioblastoma cohort with transcriptomic data, positive correlations between anti-inflammatory microglia/macrophage markers (triggering receptor expressed on myeloid cells 2, CD163 and CD32a) and CD4^+^/CD8^+^/programmed death-ligand 1 RNA expression were validated (*P* < 0.001). Finally, multivariate analysis showed that high triggering receptor expressed on myeloid cells 2, programmed death-ligand 1 and CD32a expression at the leading edge were significantly associated with poorer overall patient survival (hazard ratio = 2.05, 3.42 and 2.11, respectively), independent of clinical variables. In conclusion, anti-inflammatory microglia/macrophages, CD8^+^ T cells and programmed death-ligand 1 are correlated in the invasive margins of glioblastoma, consistent with immune-suppressive interactions. High triggering receptor expressed on myeloid cells 2, programmed death-ligand 1 and CD32a expression at the human glioblastoma leading edge are predictors of poorer overall survival. Given substantial interest in targeting microglia/macrophages, together with immune checkpoint inhibitors in cancer, these data have major clinical implications.

## Teaser

Noorani *et al.* investigated the immune-phenotype of glioblastoma cores and their invasive margins. They demonstrated reduced macrophage/microglial activation at the tumour margins and a correlation between anti-inflammatory macrophages and CD8^+^ T-cell infiltration at the tumour margin. High triggering receptor expressed on myeloid cells 2, programmed death-ligand 1 and CD32a expression predicted poorer survival in glioblastoma.

## Graphical Abstract

**Graphical Abstract:**
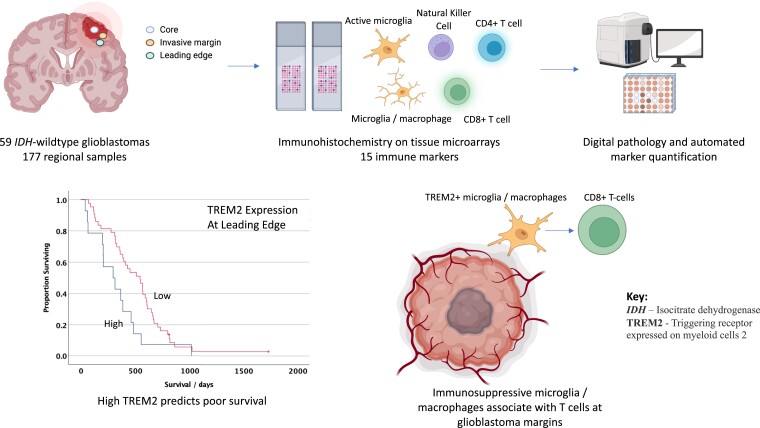

## Introduction

Glioblastoma is the most frequent primary intrinsic brain tumour in adults and carries a devastating prognosis with a median survival of only 14 months with standard-of-care treatment.^1^ Unlike the improvements in clinical outcomes due to targeted treatments in other types of cancer, such agents have so far not resulted in meaningful improvements in survival in patients with glioblastoma.^2-4^ Immune-based therapies targeting either tumour-associated macrophages or microglia (TAMs) or the adaptive immunity for glioblastoma are promising,^5^ with a large number of immunotherapy trials in progress.^6-9^ However, recent negative early phase trials highlight the need for improved stratification of patients in order to yield meaningful outcomes.^4^

Glioblastoma is a molecularly heterogeneous cancer with an immunosuppressive tumour microenvironment (TME).^10-12^ Brain-resident microglia and infiltrating macrophages constitute a large fraction (up to 50%) of cells within a glioblastoma.^13-15^ Single-cell RNA-sequencing studies have recently revealed substantial heterogeneity in the transcriptional phenotypes of microglia and T cells, with a high proportion of exhausted T cells.^16,17^ The T-cell receptor with immunoglobulin (Ig) and ITIM domains (TIGIT) is a marker of T-cell exhaustion, and TIGIT is highly expressed in glioblastoma-infiltrating T cells.^18^ TAMs are correlated with an increased grade of gliomas^19^ and typically show an anti-inflammatory^20^ (and protumourigenic) rather than a proinflammatory cytokine profile in glioblastoma;^21-23^ hence, TAM-targeting therapies are being explored in glioblastoma.^24,25^ Emerging studies indicate that interactions between microglia/macrophages and T cells may play a dominant role in driving T-cell dysfunction and hence the absence of anti-tumour immunity in glioblastoma and other cancers,^17,26,27^ fuelling tumour growth. For example, recent pre-clinical modelling demonstrates that triggering receptor expressed on myeloid cells 2 (TREM2) expression in TAMs in several cancers is associated with T-cell exhaustion and anti-PD1 therapy resistance,^28,29^ and TREM2 antibody therapy is being investigated in cancers with TAMs expressing this molecule,^30^ although TREM2 relevance in human glioblastoma is yet to be established.

Programmed death-ligand 1 (PD-L1) binds to its receptor PD-1 and CD80 to suppress T-cell-mediated cancer cell killing by restricting T-cell effector functions.^31-33^ Two mechanisms are proposed for the regulation of PD-L1 by tumour cells: adaptive immune resistance, in which PD-L1 is upregulated on tumour cells in response to interferon gamma signalling from CD8^+^ T cells, and intrinsic immune resistance whereby PD-L1 expression in tumour cells is elicited by oncogenic signalling. Anti-PD1 (immune checkpoint inhibitor) therapies are more likely to be effective in tumours with PD-L1 suppressing adaptive immunity,^34^ although regional heterogeneity in PD-L1 expression has not been fully characterized in glioblastoma.

The ability of glioblastoma cells to infiltrate into the surrounding brain renders surgical resection ineffective at removing all cancer cells. In spite of 90% of recurrences taking place at the glioblastoma invasive margins, only a handful of studies have focused on the immune microenvironment in this area. It is becoming apparent that glioblastomas harbour substantial spatial heterogeneity,^35^ with the tumour core and invading margins having different stem-cell populations and kinase signalling activities.^36,37^ Analysis of the glioblastoma TME is typically limited to a single region of the tumour core, which precludes detection of spatial variation in TAM/T-cell phenotypes. Emerging data point towards substantial spatial heterogeneity in the expression of immune-related proteins, such as in areas of normoxia compared with those of hypoxia.^38^ Deeper understanding of the spatial heterogeneity of the glioblastoma TME, including interactions between T cells and microglia/macrophages, will be essential for harnessing immune-based therapies for the benefit of patients with glioblastoma.^39-41^

Given major interest in targeting TAMs across cancers, potentially in combination with immune checkpoint inhibitors,^29^ we investigated the immunophenotype of microglia/macrophages, natural killer (NK) and T cells by immunostaining for 15 markers in glioblastomas from 59 patients. We examined three key regions per tumour, including the core, infiltrating zone and leading edge. We were able to identify the functional phenotypes of TAMs and their relationships with T/NK cells in these different regions, as well as their impact on clinical outcomes.

## Materials and methods

### Cases

This retrospective analysis included biopsies of 59 consecutive patients with newly diagnosed glioblastoma (*IDH1*-wild type, WHO Grade 4, according to 2016 WHO classification) treated at University Hospital Southampton between March 2017 and June 2020; surgical tissue specimens were obtained from the archives of the Department of Cellular Pathology, University Hospital Southampton, UK. For routine diagnostic purposes, *IDH1* mutation status was defined by immunohistochemistry with an antibody specific for the common *IDH1* mutation (R132H) supplemented with isocitrate dehydrogenase ½ (*IDH1/2*) gene sequencing as appropriate. All tumours had retained nuclear α-thalassemia/mental-retardation-syndrome-X-linked immunoreactivity.^42^

Tissue microarray (TMA) paraffin blocks were generated using these surgical specimens. Three tumour regions per patient were sampled for the TMAs: (i) tumour core, representing a solid tumour and avoiding as far as possible tumour necrosis and microvascular proliferation; (ii) infiltrating zone, where tumour cells are invading into brain parenchyma, which retains its essential structure; and (iii) leading edge, representing the most ‘normal’ brain tissue in the specimen, mostly comprising cortical grey matter and lacking significant numbers of morphologically detectable tumour cells (*n* = 177 samples). Only one sample from each of these regions was studied per patient. Differentiation between the infiltrating zone and leading edge was performed based on histology from the same biopsy sample by two independent neuropathologists.

The clinical details of the patients are shown in Supplementary Table 1. Patients underwent either complete gross or partial excision of their tumour (as defined intraoperatively), with the extent of resection largely determined by the proximity of the tumour to eloquent regions. The mean age of these patients was 61.6 years (range: 38–80 years); 36 patients were male and 22 were female. Of the 59 glioblastomas, 32 displayed *MGMT* (O(6)-methylguanine-DNA methyltransferase) methylation. Patients were routinely started on dexamethasone with gastroprotection preoperatively for up to 1 week, and this was then tapered off postoperatively. Patients had an MRI head scan postoperatively with contrast within 72 h to assess any residual tumour. Patients underwent whole-brain therapy and treatment with temozolomide after they recovered from surgery. Two patients were lost to follow-up and not included in survival analyses. Three patients underwent re-resection following a tumour recurrence; these recurrences were not analysed in this study. All deaths in this cohort were due to unfortunately aggressive course of the underlying glioblastoma.

### Ethics

Our study received ethical approval from the UK Brain Archive Information Network (BRAIN UK),^43^ Research Ethics Committee South Central Hampshire B (REC reference number 14/SC/0098).

### Immunohistochemistry

Six micrometre sections of formalin-fixed paraffin-embedded tissue from the TMA blocks were immunostained. The appropriate antigen retrieval steps were performed prior to addition of the primary antibodies. The list of primary antibodies used in the study is presented in Supplementary Table 2. Visualization of biotinylated secondary antibodies (Dako, Denmark) was achieved by using the avidin–biotin–peroxidase complex method (Vectastain Elite) with 3,3′-diaminobenzidine as the chromogen (Vector Laboratories, UK). The TMA sections were then counterstained with haematoxylin, dehydrated and mounted on DePeX (VWR International, UK). Appropriate negative controls were performed to ensure the specificity of the staining.

### Quantification

The TMAs were scanned at magnification × 20 using the Olympus dotSlide system and TMA software (Olympus, UK). Digital image analysis of the immunostaining was performed with Fiji/ImageJ (Wayne Rasband, National Institutes of Health, Bethesda, MD, USA, version 1.49u^44^) to obtain protein load. For each antibody, a threshold was determined to quantify the percentage image area immunostained by the antibody and expressed as protein load (%). The use of protein load as the standard quantification measure for all immunostaining in human glioma samples provided consistency within the measurement allowing fair comparisons between regions.

### Statistical analysis

The normality of the data was assessed by the Shapiro–Wilk test and the assessment of quantile–quantile plots. For comparison between tumour regions, one-way ANOVA or a Kruskal–Wallis test were used for normally and non-normally distributed data, respectively, with Bonferroni correction for multiple testing; alpha was pre-set at 0.05. To determine relationships between markers, the Spearman’s rank correlation coefficient was analysed, given that the data were non-parametric; *P* < 0.01 was taken as the threshold for statistical significance. All hypotheses were two tailed. Kaplan–Meier curves were plotted to assess the association between immune markers and overall patient survival; for plotting survival curves, marker expressions as continuous data were converted to categorical variables, with the upper quartile being used as the cut-off for ‘high’ versus ‘low’ expression. Cases were censored if the patients were alive at the most recent clinic visit. The overall survival time was deemed to be the time from first diagnosis to death. Survival curves were compared by the log-rank test and *P*-values <0.05 were taken to be statistically significant. As clinical features may be prognostic for glioblastoma,^45,46^ the effect of clinical variables (age >65 years, sex, complete versus subtotal resection, *MGMT* methylation status) on overall survival was assessed using Cox regression analysis. For clinical variables where univariate Cox regression analysis showed a significant effect or statistical trend (*P* < 0.1), a multivariate Cox proportional hazards analysis with the relevant immune marker and clinical variable was performed to obtain a hazard ratio [HR; with 95% confidence interval (CI)] for overall survival, with *P* < 0.05 being taken as the statistical significance threshold. Statistical analyses were performed with IBM SPSS Statistics (SPSS Inc., Chicago, IL, USA).

### The Cancer Genome Atlas analysis

Patient survival data and tumour gene expression data (Affymetrix microarray) from The Cancer Genome Atlas (TCGA) glioblastoma data set were analysed through the Betastasis software (www.betastasis.com). A *P*-value of <0.05 from the log-rank test was considered to be statistically significant when comparing survival curves; cases were stratified according to whether marker expression was above or below the median (high and low, respectively).

## Results

### Microglial/macrophage markers reveal anti-inflammatory populations

**Figure 1:**
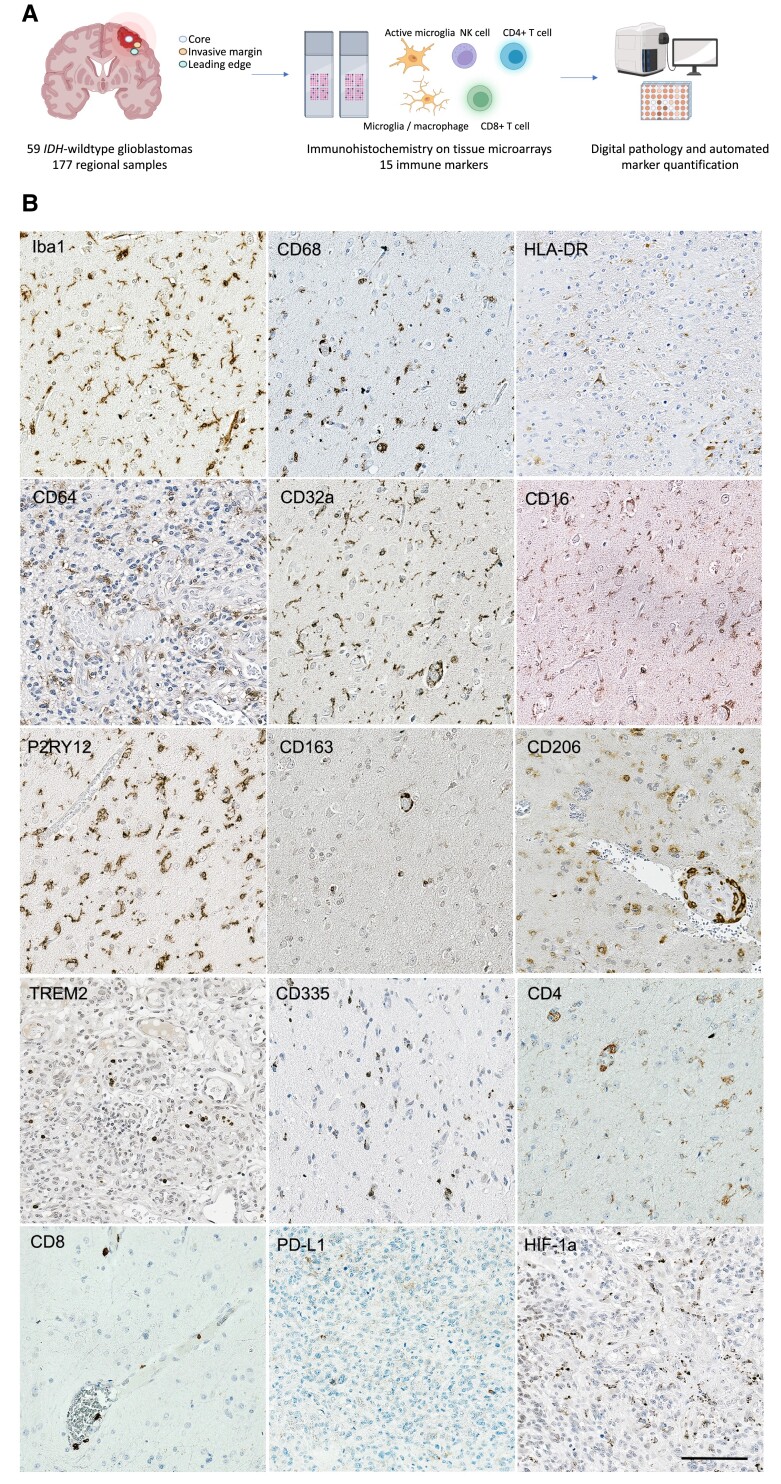
**Experimental outline for immune phenotyping of human glioblastomas.** (**A**) Experimental workflow for this study. (**B**) Representative examples of immunostaining for 15 markers including microglial/macrophage phenotypes, NK cells (CD335), CD4^+^ and CD8^+^ T cells, PD-L1 and HIF-1α. Counterstaining is with haematoxylin. Scale bar = 20μm.

Several markers associated with different microglial and macrophage functions were investigated: HLA-DR is a Major Histocompatibility Class II cell-surface receptor responsible for antigen presentation for non-self-recognition,^47^ and can mark proinflammatory brain TAMs;^48^ Iba1 is a marker of microglial motility and migration;^49^ P2RY12 is a purinergic receptor specifically expressed in homeostatic microglia;^50,51^ CD68 is linked to phagocytosis as an endosomal/lysosomal-associated transmembrane glycoprotein;^52,53^ and TREM2, which is associated with blood-derived infiltrative macrophages^54,55^ and has recently been demonstrated to be expressed on a subset of anti-inflammatory TAMs in several cancer types.^28,56^ CD163 and CD206 are more specific for perivascular macrophages in the brain and associated with an anti-inflammatory profile.^48^ We studied the central effectors of IgG-mediated immune responses, FcγRs, by using markers for CD64 (FcgRI), a high-affinity-activating receptor for the Fc portion of IgG; CD32a (FcgRIIa) and CD16 (FcgRIII), which are low/medium-affinity-activating receptors of microglia/macrophages responding to immune complexes.^57^ CD32a is considered to be a marker of phagocytic and anti-inflammatory microglia.^20^ CD335 was used as a marker of the NK cells. Hypoxia-inducible factor 1α (HIF-1α) immunostaining revealed areas of tumour cells under hypoxic conditions particularly in regions of abnormal vasculature (Fig. 1). PD-L1 expression in tumour cells was heterogeneous across tumour core samples.

We found HLA-DR, Iba1, CD68, TREM2, P2RY12, CD64, CD16 and CD32a immunolabelled tumour-associated microglia and perivascular macrophages in the tumour cores. Iba1-, CD32a- and CD163-expressing TAMs were abundant in the glioblastoma cores (protein load 6.11, 7.41 and 3.09%, respectively). Of note, there was a strikingly low level of HLA-DR expression (protein load 1.30%). The markers CD163 and CD206 largely immunolabelled perivascular TAM populations. These data imply that TAMs largely show an activated and anti-inflammatory phenotype in the glioblastoma core.

### Regional TAM and T-cell analysis reveals spatial heterogeneity

**Figure 2:**
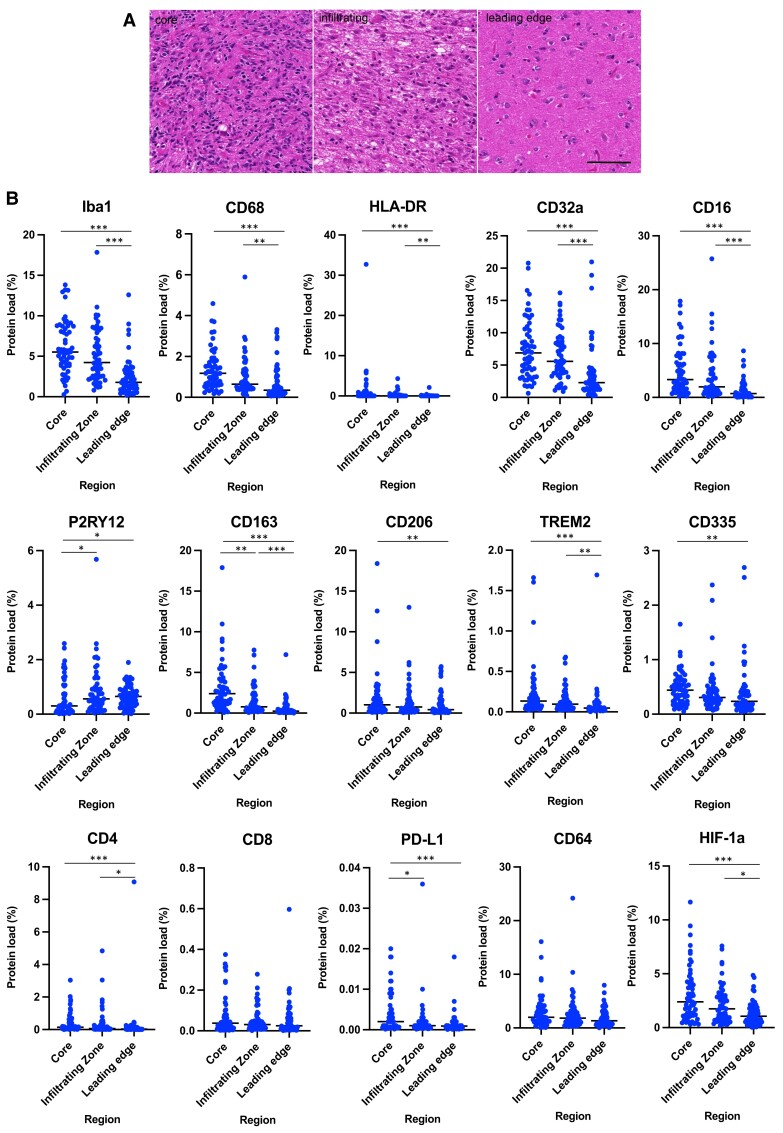
**Quantitative evaluation of microglia/macrophages expressing different phenotypic markers, HIF-1α, PD-L1, NK and T cells across regions of 59 human glioblastomas.** (**A**) Representative haematoxylin and eosin–stained images from the core, infiltrating zone and leading edge of a glioblastoma. The core contains typical histological appearances of a glioblastoma, the infiltrating zone contains diffuse hypercellularity as tumour cells invade surrounding brain, and the leading edge contains mostly histologically ‘normal’ brain tissue surrounding the tumour mass. (**B**) After immunohistochemistry for these markers, the protein load for each marker was quantified. TAM motility and activation (Iba1, CD68) is reduced in the tumour margins compared with core; this is also the case for the proinflammatory marker HLA-DR, low-affinity-activating receptors of TAMs (CD16, CD32a), and anti-inflammatory and perivascular TAM markers (CD163, TREM2, CD206). P2RY12, the marker associated with the homeostatic state of microglia, is increased in the tumour margins compared with the core. NK cells (CD335), CD4^+^ T cells and PD-L1 are reduced in the tumour margins versus the core, whereas there was no significant difference in CD8^+^ T cells or CD64 (receptor required for autoreactive T-cell responses) between regions. HIF-1α, a marker for tumour hypoxia, is higher in the glioblastoma core compared with the margins. Each data point represents the marker protein load for one patient. Dashed line indicates the mean protein load. Kruskal–Wallis test with Bonferroni correction; *P < 0.05, **P < 0.01, ***P < 0.001.

We examined macrophage/microglial markers across three spatially distinct regions for each glioblastoma: the tumour core, infiltrating zone and leading edge. The microglia markers Iba1, CD68, HLA-DR and anti-inflammatory TAM marker TREM2 loads were significantly lower in the leading edge compared with their corresponding levels in the infiltrating zone and core (*P* < 0.05, Kruskal–Wallis test; Supplementary Table 3 and Fig. 2). In contrast, P2RY12, a marker of homeostatic microglia, was significantly reduced in the core compared with the infiltrating zone and leading edge (*P* < 0.05), implying loss of homeostatic microglia in the glioblastoma core. Although there was no difference in CD64 expression between the tumour regions, the low-affinity-activating receptors CD32a and CD16 had significantly lower expression in the leading edge compared with the infiltrating zone and core (*P* < 0.001). CD163 was significantly higher in the core compared with the infiltrating zone and leading edge (*P* = 0.002 core versus infiltrating zone; *P* < 0.001 core versus leading edge); similarly, CD206, which also labels perivascular macrophages, was lower in the leading edge compared with the core (*P* = 0.008). HIF-1α expression was significantly reduced in the leading edge of the glioblastomas compared with the infiltrating zone and core (*P* < 0.001 core versus leading edge; *P* = 0.029 infiltrating zone versus leading edge), suggesting a lower level of hypoxia in the leading edge.

PD-L1 was higher in the core compared with the infiltrating zone and leading edge of the tumours (*P* = 0.037 and *P* < 0.001, respectively). CD335, a marker for natural killer cells, was higher in the core compared with the leading edge (*P* = 0.003; Supplementary Table 3). When analysing T-cell markers, we observed that although there was no significant difference in the level of CD8^+^ T cells across the three different regions, CD4^+^ T cells were decreased in the leading edge compared with the core and infiltrating zone (*P* < 0.001 and *P* = 0.012, respectively).

Overall, these data highlight the substantial regional heterogeneity in immune cell composition and phenotypes across glioblastomas, particularly in regard to differences between the invasive margins and the tumour core.

### Associations between TAM, T- and NK-cell populations

**Figure 3:**
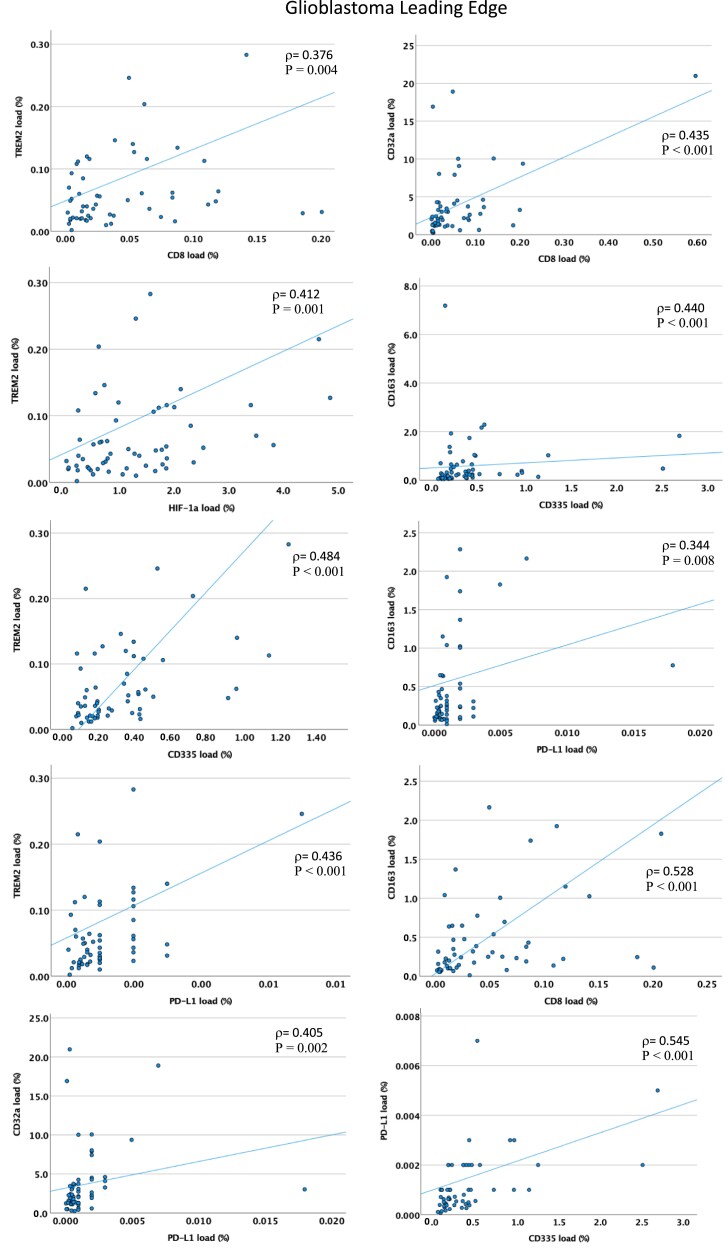
**Relationships between phenotypes of tumour-associated microglia/macrophages and adaptive immune cells in the leading edge (tumour margin) of glioblastomas.** TREM2, an anti-inflammatory TAM marker, is positively correlated with CD8^+^ T cells, NK cells (CD335) HIF-1α and PD-L1. CD32a, a low-affinity-activating receptor and marker of anti-inflammatory TAMs, is also positively correlated with CD8^+^ T cells and PD-L1 in the leading edge of these 59 glioblastomas. CD163, another anti-inflammatory and perivascular TAM marker, similarly correlates with CD8^+^ T cells, PD-L1 and NK cells (CD335). PD-L1 also positively correlates with NK cells (CD335). Each data point represents the protein load (%) for a single patient. ρ = Spearman’s rank correlation coefficient; *P* < 0.01 in all cases.

To uncover putative interactions between the different immune markers, we conducted an association analysis for the different glioblastoma regions. In all three regions, there were significant positive associations between the microglial markers Iba1, CD32a and CD16 (*P* < 0.001, Spearman’s rank test). There was a positive association between CD4^+^ T-cell infiltration and the microglial markers Iba1 and CD68 in all three areas (*P* < 0.001; Supplementary Table 4). CD8^+^ T cells were positively associated with TAM phagocytic marker CD68 in the infiltrating zone and leading edge (*P* < 0.001), but not in the tumour core. Expression of the anti-inflammatory TAM marker TREM2 was positively correlated with CD4^+^ and CD8^+^ T-cell infiltration in the leading edge and infiltration zone (*P* < 0.01) but not in the core, Fig. 3 and Supplementary Table 4. The anti-inflammatory marker CD163 was positively associated with CD8^+^ T cells in all tumour regions (*P* < 0.01, in all cases). These findings suggest there are functional interactions between anti-inflammatory TAMs and CD4^+^/CD8^+^ T cells in all regions, and in the case of CD8^+^ T cells, particularly in the tumour margins.

Notably, there was an absence of a relationship between CD64 (a receptor required for autoreactive T-cell responses) and CD8^+^/CD4^+^ T cells in all areas. There was also an absence of a relationship between the antigen presentation/proinflammatory TAM marker HLA-DR and microglial motility (Iba1) in the infiltrating zone and leading edge, although there was a positive association in the core. The hypoxia marker HIF-1α was positively associated with TAM low-affinity-activating receptor CD32a and with TREM2 (anti-inflammatory TAM marker) in all regions (*P* < 0.01). However, there was no association between HIF-1α and CD64 or HLA-DR in any area. These results implicate a microglial defect in antigen presentation in tumour margins, and a defective ability to activate functional T-cell responses in all tumour areas, with an anti-inflammatory TAM phenotype linked with tumour hypoxia.

We observed a significant positive association between PD-L1 and TAM markers (including anti-inflammatory markers), CD68, CD163, CD32a and TREM2 in the leading edge (*P* < 0.01), but not in the core or infiltrating zone (Fig. 3). Similarly, there was a positive association between PD-L1 expression and CD8^+^ T-cell infiltration in the leading edge only (*P* < 0.001), supporting the concept that immune suppression of T cells via PD-L1 mechanisms is present in the leading edge and may be driven in part by anti-inflammatory TAMs.

CD335 was positively associated with PD-L1 at both the leading edge and infiltrating zone of glioblastomas (*P* < 0.01) but not in the core; CD335 also positively correlated with CD8^+^ T cells as well as with CD68/CD163/TREM2 TAMs only at the leading edge (*P* < 0.01). These data imply anti-inflammatory TAMs may further contribute to the immune-suppressive TME at the invasive margins by dampening NK-cell effector functions.

### TREM2, Pd-L1 and CD32a correlate with patient survival

**Figure 4:**
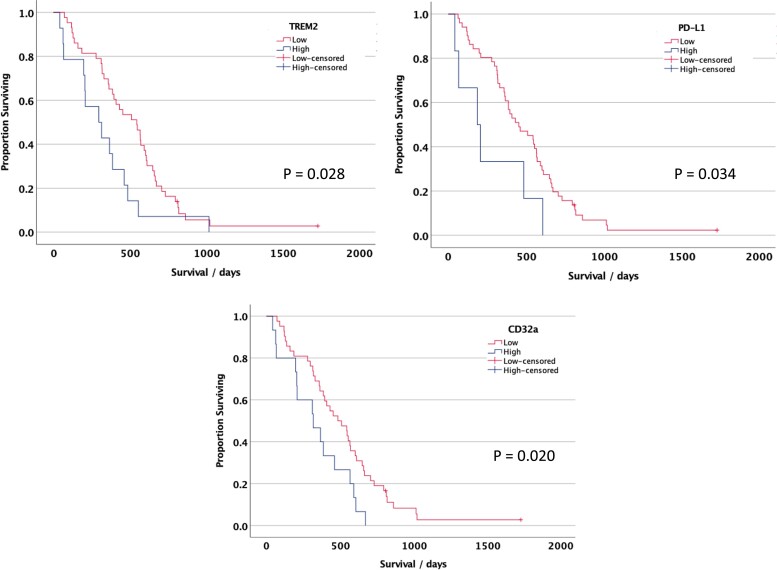
**Kaplan–Meier survival curves for prognostic leading edge markers in human glioblastoma.** Patients whose glioblastomas had a high TREM2 (**A**), CD32a (**B**) or PD-L1 (**C**) at the leading edge had a significantly shorter overall survival compared with those with low TREM, CD32a or PD-L1 (*P* < 0.05, log-rank test); *n* = 57 patients. The cut-off threshold was the upper quartile for expression of each marker.

**Figure 5:**
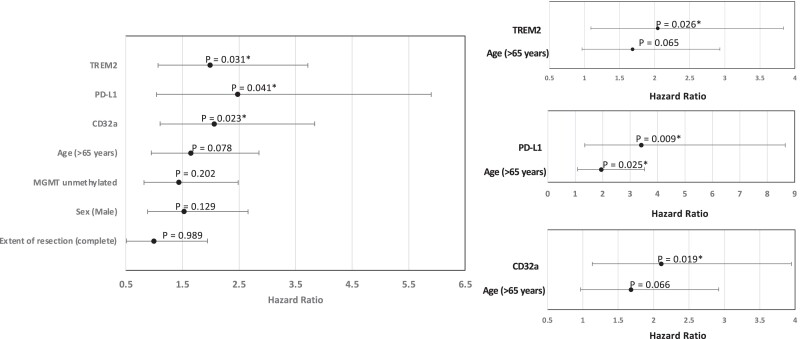
**Relationship between TREM2, PD-L1 and CD32a expression and overall survival in glioblastoma.** (**A**) Forest plot showing the hazard ratios and 95% confidence intervals for these factors and clinical variables. (**B**) Forest plot showing results of multivariate Cox regression analysis with each immune marker and patient age. *P < 0.05.

We next examined whether the TAM, NK- or T-cell markers correlate with glioblastoma patient survival in this cohort. When stratified by the tumour core or infiltration zone, none of the markers were significantly associated with overall patient survival. Analysing TAM markers in the leading edge of the tumours revealed high CD32a, TREM2 or PD-L1 was associated with poorer overall survival of glioblastoma patients (log-rank test *P* = 0.020, 0.028, 0.034, respectively, for the top quartile versus lower quartiles of each marker; Fig. 4). Certain clinical variables have been linked with outcome in glioblastoma, for example, sex-specific differences are increasingly recognized.^58^ In our cohort, none of the clinical variables (age, sex, *MGMT* methylation status and complete versus subtotal resection) showed an association with survival in univariate Cox regression analysis, except there was a trend for poorer survival with age >65 years (*P* = 0.078, HR = 1.64, CI 0.95–2.86; Fig. 5A). In a multivariate Cox proportional hazards analysis, including age and TREM2, PD-L1 or CD32a expression, each of these markers remained prognostic for poorer survival (TREM2: *P* = 0.026, HR = 2.05, CI 1.09–3.84; PD-L1: *P* = 0.009, HR = 3.42, CI 1.35–8.65; CD32a: *P* = 0.019, HR = 2.11, CI 1.13–3.95; Fig. 5B).

These findings highlight that the regional differences observed in the immunophenotypes in glioblastoma are clinically relevant and that high TREM2/CD32a/PD-L1 expression in the leading edge of glioblastomas portends a poor prognosis independent of clinical variables.

### Validation with an independent clinical cohort

**Figure 6:**
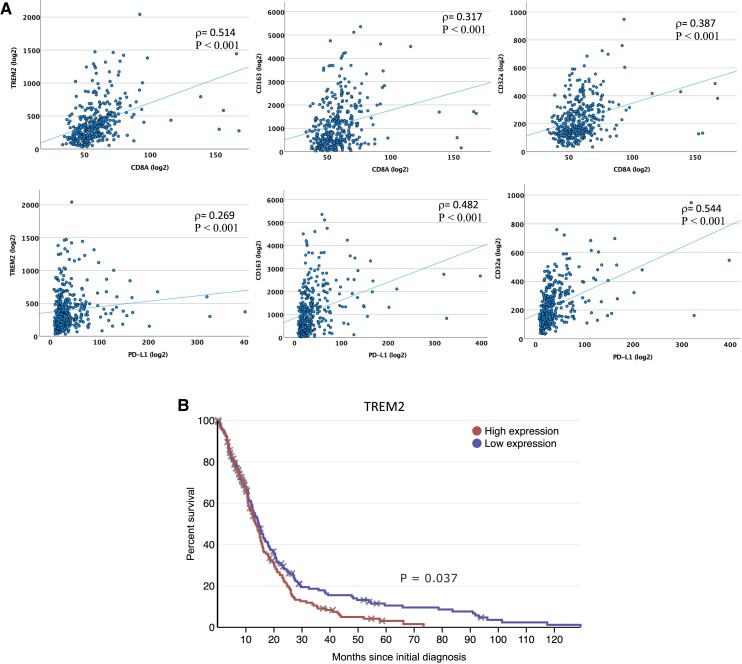
**Validation of prognostic relevance of TREM2, and the relationships between anti-inflammatory TAMs and PD-L1/CD^+^ T cells in an independent glioblastoma cohort.** (**A**) In TCGA transcriptomic data, TREM2 (anti-inflammatory TAM marker) expression positively correlated with CD8A and PD-L1 expression; similarly, CD163 (anti-inflammatory TAM marker) and CD32a (low-affinity-activating receptor and anti-inflammatory TAM marker) positively correlated with CD8a and PD-L1. ρ = Spearman’s rank correlation coefficient; *P* < 0.001 in all cases. (**B**) In TCGA glioblastoma cohort with bulk transcriptomic data, Kaplan–Meier analysis revealed that patients with glioblastomas containing high RNA expression (above median) of TREM2 had significantly poorer overall survival compared with those with low expression (*P* = 0.037, log-rank test); *n* = 349 patients.

To confirm the validity of our findings, we analysed bulk transcriptomic data from the TCGA cohort (*n* = 356 glioblastoma patients^11,59^) for immune phenotypic markers, in particular anti-inflammatory TAM and T-cell markers. This analysis confirmed a significant positive association between TREM2 and CD4^+^/CD8^+^/PD-L1; CD163 and CD4^+^/CD8^+^/PD-L1; and CD32a and CD4^+^/CD8^+^/PD-L1 (*P* < 0.001, Spearman’s rank test; Fig. 6A). In keeping with our cohort, Kaplan–Meier analysis of survival times in this independent cohort revealed that high TREM2 expression was associated with a reduced survival time compared with low TREM2 (*P* = 0.037, log-rank test; Fig. 6B). However, there was no significant association between high PD-L1 or CD32a expression and survival time in TCGA. Although this analysis is from bulk RNA data compared with protein expression and is from global rather than regional tumour samples, these data independently support findings in our patient cohort implicating a relationship between anti-inflammatory TAMs and immune-suppressed T cells and confirm prognostic relevance of TREM2 expression.

## Discussion

Several immunotherapy trials targeting TAMs or T cells are underway for patients with glioblastoma.^60^ Given limitations in surgical tissue availability, studies focused on the TME in glioblastoma typically analyse a single-core sample. However, this precludes analysis of the tumour invasive margins. Here, we immunophenotyped 59 glioblastomas, with 3 regions per patient (*n* = 177 samples), with 15 markers for TAMs, T cells and NK cells. We showed glioblastoma invasive margins have a different immune profile compared with the core, including reduced TAM motility/activation (Iba1, CD68), increased homeostatic microglia (P2RY12) and reduced CD4^+^ T and NK cells.^61^

Our study has several important implications for management of glioblastomas with immune-targeting therapies. First, we show for the first time that TREM2 expression in TAMs correlates with T-cell infiltration and that TREM2 and CD32a expression at the glioblastoma margin predict a poor prognosis. This highlights a role for TREM2-expressing anti-inflammatory TAMs in driving immune suppression in the TME. Given CD32a has also been associated with anti-inflammatory microglia, it is likely that the correlation between CD32a expression and poor survival is due to anti-inflammatory effects of TAMs similar to TREM2. We also noted that these anti-inflammatory TAM markers correlated with HIF-1α, suggesting this TAM phenotype is linked with tumour hypoxic regions. Apoptotic cells in hypoxic regions may be a strong attractant for TAMs.^62^ This would add to recent data showing that phagocytic TAMs populate hypoxic pseudopalisades to facilitate glioblastoma invasion.^63^

Second, we find significant associations between TAMs (Iba1, CD68, TREM2, CD32a) and CD8^+^ T cells specifically in the tumour margins rather than the tumour core, suggesting TAMs may contribute to immune suppression of CD8^+^ T cells more strongly at the invasive front of glioblastoma and that this region warrants further exploration for stratification of patients in clinical trials. This adds mechanistic insight to previous data highlighting T-cell exhaustion in glioblastoma.^18^ There was no relationship between CD64 (a receptor required for autoreactive T-cell responses) and CD8^+^/CD4^+^ T cells in any area, and no relationship between the antigen presentation/proinflammatory TAM marker HLA-DR and microglial motility (Iba1) in the tumour margins, consistent with a suppressed T-cell response that is likely due in part to a defect in TAM antigen presentation. Similarly, we observed a previously undescribed association between anti-inflammatory TAMs and NK-cell infiltration at the glioblastoma leading edge; this immunosuppressive environment is likely to dampen NK-cell effector ability to eradicate tumour cells.^64^

Third, we show that the level of PD-L1 expression correlates with CD8^+^ T-cell infiltration at the tumour margins, suggesting that an active immune resistance mechanism is present, which may potentially be overcome by PD1/PD-L1 therapy in a subset of glioblastomas with PD-L1 expression at the tumour margin. It has been suggested that PD-L1 expression, particularly at the invasive front, may be an immune-suppressive mechanism in other cancers.^65^ However, given that we have demonstrated that PD-L1 expression is generally low in most glioblastomas, other mechanisms are likely to exist for T-cell suppression, particularly in the tumour core where we observed an absence of a correlation between PD-L1 and CD8^+^ T cells.

A study employing flow-cytometry with a panel of immune markers demonstrated glioblastomas can be categorized into those with few leukocytes, those with predominantly TAMs and neutrophils, and those with a mixed myeloid and lymphoid immune infiltrate, with the latter cases with mixed infiltrates having a worse prognosis.^66^ Landry *et al.*^67^ analysed single-cell data from four patients to infer that TAMs at the edge of glioblastoma have an anti-inflammatory phenotype associated with nuclear factor kappa-light-chain-enhancer of activated B-cell signalling. Our study of a larger cohort and different techniques extends these observations by confirming an association between T cells and TAMs at the invading margins of glioblastomas, particularly in the case of anti-inflammatory TREM2^+^ TAMs, the expression of which correlated with poorer survival.

Possible mechanistic explanations behind TAM interactions with T cells include the expression of CD39 in TAMs, leading to CD8^+^ T-cell dysfunction by producing adenosine in cooperation with CD73, as observed in mouse models.^68^ The secretion of immune-suppressive cytokines such as IL-10 and transforming growth factor-β by microglia can also drive this phenotype in T cells.^69^ We hypothesize that a highly immune-suppressed TME at the tumour margins is likely to facilitate invasion into the surrounding brain by enabling cancer cells to evade destruction. Given emerging discoveries on neuronal—tumour interactions,^70^ it is possible that surrounding neurons may support TAM—T-cell interactions at the tumour-brain interface.

Based on the recent developments in TREM2-targeting antibodies, the results of this study may warrant trialling of combination therapy consisting of anti-TREM2 and anti-PD-L1 therapy in glioblastomas expressing these molecules. Functional and detailed molecular studies *in vivo* should provide deeper insight into the mechanisms underlying the TAM and T-cell interactions at the tumour margins compared with the core.^71^ Faithful glioblastoma mouse models that reflect human phenotypes will be invaluable for this.^72^

### Limitations and further approaches

Our study investigated patients with newly diagnosed glioblastoma only. Evidence suggests treatment with temozolomide and radiotherapy affects the glioblastoma TME, including PD-L1 expression;^73^ and so analysis of tumours after treatment in multiple regions as done here is warranted in future. Functional experiments will be needed to determine whether the TAM phenotype observed at the leading edge of glioblastoma helps induce tumour cell PD-L1 expression. Our staining experiments employed brightfield single-antibody labelling rather than fluorescent double labelling, which has more limited power for detecting other proteins in the tissue but allows for a better assessment of cells in the tissue environment. Further studies would allow subcategorization of glioblastomas by molecular features, including transcriptional subtypes and mutations/copy number changes, to determine how our findings apply to these different subtypes.^74^ For example, in a cohort of 98 patients, the subtype of mesenchymal glioblastomas was found to have the most infiltration of microglia, macrophages and lymphocytes.^75^ As extent of resection influences survival,^76^ a detailed volumetric analysis of residual tumour integrated with TME profiling is warranted too. Such additional work may generate more nuanced knowledge of the glioblastoma TME and the interactions between anti-inflammatory TAMs, CD4^+^/CD8^+^ T cells and immune suppression, and is likely to yield novel therapeutic targets.

## Conclusion

Compared with the glioblastoma core, the invasive margins have reduced microglial activation and motility, CD4^+^ T and NK cells, and have increased homeostatic microglia. Anti-inflammatory TAMs, T cells and PD-L1 are positively correlated in the invasive margins of human glioblastoma, suggesting functional interactions for suppression of anti-tumour immunity. Anti-inflammatory TAM markers (TREM2 and CD32a) are linked with tumour hypoxia (HIF-1α). High TREM2, PD-L1 and CD32a expressions at the tumour leading edge are predictors of poorer overall survival, independent of clinical factors. Currently, only the core samples of glioblastoma are routinely analysed with histopathology, and there is a clinical need for more prognostic biomarkers. The results from this study should prompt an analysis of both the core and the margins of glioblastomas to potentially aid clinical prognostication, in particular TREM2 immunohistochemical quantification; and our data suggest that trialling combination TAM-targeting therapy and immune checkpoint inhibitors may be warranted in a subgroup of these patients.

## Supplementary Material

fcad176_Supplementary_Data

## Data Availability

The data that support the findings of this study are not publicly available, in order to maintain the confidentiality of the patients. The data are, however, available from the corresponding author upon reasonable request.

## Abbreviations

BRAIN UK =: UK Brain Archive Information Network
HIF-1α =: hypoxia-inducible factor 1α
Ig =: immunoglobulin
*MGMT* =: O(6)-methylguanine-DNA methyltransferase
NK =: natural killer
PD-L1 =: programmed death-ligand 1
TAMs =: tumour-associated macrophages or microglia
TCGA =: The Cancer Genome Atlas
TIGIT =: T-cell receptor with Ig and ITIM domains
TMA =: tissue microarray
TME =: tumour microenvironment
TREM2 =: triggering receptor expressed on myeloid cells 2

## References

[1] Stupp R, Mason WP, van den Bent MJ, et al Radiotherapy plus concomitant and adjuvant temozolomide for glioblastoma. N Engl J Med. 2005;352(10):987–996. 15758009 10.1056/NEJMoa043330

[2] Weller M, Butowski N, Tran DD, et al Rindopepimut with temozolomide for patients with newly diagnosed, EGFRvIII-expressing glioblastoma (ACT IV): A randomised, double-blind, international phase 3 trial. Lancet Oncol. 2017;18(10):1373–1385. 28844499 10.1016/S1470-2045(17)30517-X

[3] Wick W, Puduvalli VK, Chamberlain MC, et al Phase III study of enzastaurin compared with lomustine in the treatment of recurrent intracranial glioblastoma. J Clin Oncol. 2010;28(7):1168–1174. 20124186 10.1200/JCO.2009.23.2595 PMC2834468

[4] Noorani I, Mischel PS, Swanton C. Leveraging extrachromosomal DNA to fine-tune trials of targeted therapy for glioblastoma: Opportunities and challenges. Nat Rev Clin Oncol. 2022;19(11):733–743. 36131011 10.1038/s41571-022-00679-1 PMC13271662

[5] Quail DF, Bowman RL, Akkari L, et al The tumor microenvironment underlies acquired resistance to CSF-1R inhibition in gliomas. Science. 2016;352(6288):aad3018. 27199435 10.1126/science.aad3018 PMC5450629

[6] Touat M, Idbaih A, Sanson M, Ligon KL. Glioblastoma targeted therapy: Updated approaches from recent biological insights. Ann Oncol. 2017;28(7):1457–1472. 28863449 10.1093/annonc/mdx106 PMC5834086

[7] Weenink B, French PJ, Sillevis Smitt PAE, Debets R, Geurts M. Immunotherapy in glioblastoma: Current shortcomings and future perspectives. Cancers (Basel). 2020;12(3):751. 32235752 10.3390/cancers12030751 PMC7140029

[8] Westphal M, Maire CL, Lamszus K. EGFR as a target for glioblastoma treatment: An unfulfilled promise. CNS Drugs. 2017;31(9):723–735. 28791656 10.1007/s40263-017-0456-6 PMC5573763

[9] Seystahl K, Wick W, Weller M. Therapeutic options in recurrent glioblastoma—An update. Crit Rev Oncol Hematol. 2016;99:389–408. 26830009 10.1016/j.critrevonc.2016.01.018

[10] Nduom EK, Weller M, Heimberger AB. Immunosuppressive mechanisms in glioblastoma. Neuro Oncol. 2015;17(Suppl 7):vii9–vii14. 26516226 10.1093/neuonc/nov151 PMC4625890

[11] Brennan CW, Verhaak RG, McKenna A, et al The somatic genomic landscape of glioblastoma. Cell. 2013;155(2):462–477. 24120142 10.1016/j.cell.2013.09.034 PMC3910500

[12] Noorani I, de la Rosa J, Choi YH, et al PiggyBac mutagenesis and exome sequencing identify genetic driver landscapes and potential therapeutic targets of EGFR-mutant gliomas. Genome Biol. 2020;21(1):181. 32727536 10.1186/s13059-020-02092-2 PMC7392733

[13] Darmanis S, Sloan SA, Croote D, et al Single-cell RNA-seq analysis of infiltrating neoplastic cells at the migrating front of human glioblastoma. Cell Rep. 2017;21(5):1399–1410. 29091775 10.1016/j.celrep.2017.10.030 PMC5810554

[14] Chen Z, Hambardzumyan D. Immune microenvironment in glioblastoma subtypes. Front Immunol. 2018;9:1004. 29867979 10.3389/fimmu.2018.01004 PMC5951930

[15] Wei J, Chen P, Gupta P, et al Immune biology of glioma-associated macrophages and microglia: Functional and therapeutic implications. Neuro Oncol. 2020;22(2):180–194. 31679017 10.1093/neuonc/noz212 PMC7442334

[16] Woroniecka K, Chongsathidkiet P, Rhodin K, et al T-cell exhaustion signatures vary with tumor type and are severe in glioblastoma. Clin Cancer Res. 2018;24(17):4175–4186. 29437767 10.1158/1078-0432.CCR-17-1846 PMC6081269

[17] Ravi VM, Neidert N, Will P, et al T-cell dysfunction in the glioblastoma microenvironment is mediated by myeloid cells releasing interleukin-10. Nat Commun. 2022;13(1):925. 35177622 10.1038/s41467-022-28523-1 PMC8854421

[18] Lucca LE, Lerner BA, Park C, et al Differential expression of the T-cell inhibitor TIGIT in glioblastoma and MS. Neurol Neuroimmunol Neuroinflamm. 2020;7(3):e712. 32269065 10.1212/NXI.0000000000000712 PMC7188477

[19] Noorani I, Petty G, Grundy PL, et al Novel association between microglia and stem cells in human gliomas: A contributor to tumour proliferation? J Pathol Clin Res. 2015;1(2):67–75. 27499894 10.1002/cjp2.7 PMC4858136

[20] Walker DG, Lue LF. Immune phenotypes of microglia in human neurodegenerative disease: Challenges to detecting microglial polarization in human brains. Alzheimers Res Ther. 2015;7(1):56. 26286145 10.1186/s13195-015-0139-9 PMC4543480

[21] Komohara Y, Ohnishi K, Kuratsu J, Takeya M. Possible involvement of the M2 anti-inflammatory macrophage phenotype in growth of human gliomas. J Pathol. 2008;216(1):15–24. 18553315 10.1002/path.2370

[22] Billingham C, Powell MR, Jenner KA, et al Rat astrocytic tumour cells are associated with an anti-inflammatory microglial phenotype in an organotypic model. Neuropathol Appl Neurobiol. 2013;39(3):243–255. 22631872 10.1111/j.1365-2990.2012.01283.x

[23] Sorensen MD, Dahlrot RH, Boldt HB, Hansen S, Kristensen BW. Tumour-associated microglia/macrophages predict poor prognosis in high-grade gliomas and correlate with an aggressive tumour subtype. Neuropathol Appl Neurobiol. 2018;44(2):185–206. 28767130 10.1111/nan.12428

[24] Akkari L, Bowman RL, Tessier J, et al Dynamic changes in glioma macrophage populations after radiotherapy reveal CSF-1R inhibition as a strategy to overcome resistance. Sci Transl Med. 2020;12(552):eaaw7843. 32669424 10.1126/scitranslmed.aaw7843

[25] Pyonteck SM, Akkari L, Schuhmacher AJ, et al CSF-1R inhibition alters macrophage polarization and blocks glioma progression. Nat Med. 2013;19(10):1264–1272. 24056773 10.1038/nm.3337 PMC3840724

[26] Hara T, Chanoch-Myers R, Mathewson ND, et al Interactions between cancer cells and immune cells drive transitions to mesenchymal-like states in glioblastoma. Cancer Cell. 2021;39(6):779–792.e11. 34087162 10.1016/j.ccell.2021.05.002 PMC8366750

[27] Gangoso E, Southgate B, Bradley L, et al Glioblastomas acquire myeloid-affiliated transcriptional programs via epigenetic immunoediting to elicit immune evasion. Cell. 2021;184(9):2454–2470.e26. 33857425 10.1016/j.cell.2021.03.023 PMC8099351

[28] Binnewies M, Pollack JL, Rudolph J, et al Targeting TREM2 on tumor-associated macrophages enhances immunotherapy. Cell Rep. 2021;37(3):109844. 34686340 10.1016/j.celrep.2021.109844

[29] Molgora M, Esaulova E, Vermi W, et al TREM2 modulation remodels the tumor myeloid landscape enhancing anti-PD-1 immunotherapy. Cell. 2020;182(4):886–900.e17. 32783918 10.1016/j.cell.2020.07.013 PMC7485282

[30] Khantakova D, Brioschi S, Molgora M. Exploring the impact of TREM2 in tumor-associated macrophages. Vaccines (Basel). 2022;10(6):943. 35746551 10.3390/vaccines10060943 PMC9227554

[31] Butte MJ, Keir ME, Phamduy TB, Sharpe AH, Freeman GJ. Programmed death-1 ligand 1 interacts specifically with the B7-1 costimulatory molecule to inhibit T cell responses. Immunity. 2007;27(1):111–122. 17629517 10.1016/j.immuni.2007.05.016 PMC2707944

[32] Park JJ, Omiya R, Matsumura Y, et al B7-H1/CD80 interaction is required for the induction and maintenance of peripheral T-cell tolerance. Blood. 2010;116(8):1291–1298. 20472828 10.1182/blood-2010-01-265975 PMC2938239

[33] Kowanetz M, Zou W, Gettinger SN, et al Differential regulation of PD-L1 expression by immune and tumor cells in NSCLC and the response to treatment with atezolizumab (anti-PD-L1). Proc Natl Acad Sci U S A. 2018;115(43):E10119–E10126. 30297397 10.1073/pnas.1802166115 PMC6205493

[34] Tumeh PC, Harview CL, Yearley JH, et al PD-1 blockade induces responses by inhibiting adaptive immune resistance. Nature. 2014;515(7528):568–571. 25428505 10.1038/nature13954 PMC4246418

[35] Ravi VM, Will P, Kueckelhaus J, et al Spatially resolved multi-omics deciphers bidirectional tumor-host interdependence in glioblastoma. Cancer Cell. 2022;40(6):639–655.e13. 35700707 10.1016/j.ccell.2022.05.009

[36] Ibrahim AN, Yamashita D, Anderson JC, et al Intratumoral spatial heterogeneity of BTK kinomic activity dictates distinct therapeutic response within a single glioblastoma tumor. J Neurosurg. 2019:1–12. Online ahead of print. 10.3171/2019.7.JNS191376 PMC7961807 31628288

[37] Minata M, Audia A, Shi J, et al Phenotypic plasticity of invasive edge glioma stem-like cells in response to ionizing radiation. Cell Rep. 2019;26(7):1893–1905.e7. 30759398 10.1016/j.celrep.2019.01.076 PMC6594377

[38] Petterson SA, Sorensen MD, Burton M, et al Differential expression of checkpoint markers in the normoxic and hypoxic microenvironment of glioblastomas. Brain Pathol. 2023;33(1):e13111. 36093941 10.1111/bpa.13111 PMC9836374

[39] Lemee JM, Clavreul A, Menei P. Intratumoral heterogeneity in glioblastoma: Don’t forget the peritumoral brain zone. Neuro Oncol. 2015;17(10):1322–1332. 26203067 10.1093/neuonc/nov119 PMC4578587

[40] Juliano J, Gil O, Hawkins-Daarud A, et al Comparative dynamics of microglial and glioma cell motility at the infiltrative margin of brain tumours. J R Soc Interface. 2018;15(139):20170582. 29445035 10.1098/rsif.2017.0582 PMC5832721

[41] Hide T, Komohara Y, Miyasato Y, et al Oligodendrocyte progenitor cells and macrophages/microglia produce glioma stem cell niches at the tumor border. EBioMedicine. 2018;30:94–104. 29559295 10.1016/j.ebiom.2018.02.024 PMC5952226

[42] Ebrahimi A, Skardelly M, Bonzheim I, et al ATRX immunostaining predicts IDH and H3F3A status in gliomas. Acta Neuropathol Commun. 2016;4(1):60. 27311324 10.1186/s40478-016-0331-6 PMC4910252

[43] Nicoll JAR, Bloom T, Clarke A, Boche D, Hilton D. BRAIN UK: Accessing NHS tissue archives for neuroscience research. Neuropathol Appl Neurobiol. 2022;48(2):e12766. 34528715 10.1111/nan.12766

[44] Schindelin J, Arganda-Carreras I, Frise E, et al Fiji: An open-source platform for biological-image analysis. Nat Methods. 2012;9(7):676–682. 22743772 10.1038/nmeth.2019 PMC3855844

[45] Gittleman H, Lim D, Kattan MW, et al An independently validated nomogram for individualized estimation of survival among patients with newly diagnosed glioblastoma: NRG Oncology RTOG 0525 and 0825. Neuro Oncol. 2017;19(5):669–677. 28453749 10.1093/neuonc/now208 PMC5464437

[46] Hegi ME, Diserens AC, Gorlia T, et al MGMT gene silencing and benefit from temozolomide in glioblastoma. N Engl J Med. 2005;352(10):997–1003. 15758010 10.1056/NEJMoa043331

[47] Styren SD, Civin WH, Rogers J. Molecular, cellular, and pathologic characterization of HLA-DR immunoreactivity in normal elderly and Alzheimer’s disease brain. Exp Neurol. 1990;110(1):93–104. 1698655 10.1016/0014-4886(90)90054-v

[48] Widodo SS, Dinevska M, Cuzcano L, et al Spatial analysis of the metastatic brain tumor immune and extracellular matrix microenvironment. Adv Cancer Biol-Metastasis. 2023. Advance Access published on January 7, 2023.

[49] Franco-Bocanegra DK, McAuley C, Nicoll JAR, Boche D. Molecular mechanisms of microglial motility: Changes in ageing and Alzheimer’s disease. Cells. 2019;8(6):639. 31242692 10.3390/cells8060639 PMC6627151

[50] Butovsky O, Jedrychowski MP, Moore CS, et al Identification of a unique TGF-beta-dependent molecular and functional signature in microglia. Nat Neurosci. 2014;17(1):131–143. 24316888 10.1038/nn.3599 PMC4066672

[51] Zrzavy T, Hametner S, Wimmer I, Butovsky O, Weiner HL, Lassmann H. Loss of ‘homeostatic’ microglia and patterns of their activation in active multiple sclerosis. Brain. 2017;140(7):1900–1913. 28541408 10.1093/brain/awx113 PMC6057548

[52] Minett T, Classey J, Matthews FE, et al Microglial immunophenotype in dementia with Alzheimer’s pathology. J Neuroinflammation. 2016;13(1):135. 27256292 10.1186/s12974-016-0601-z PMC4890505

[53] Rabinowitz SS, Gordon S. Macrosialin, a macrophage-restricted membrane sialoprotein differentially glycosylated in response to inflammatory stimuli. J Exp Med. 1991;174(4):827–836. 1919437 10.1084/jem.174.4.827 PMC2118958

[54] Klesney-Tait J, Turnbull IR, Colonna M. The TREM receptor family and signal integration. Nat Immunol. 2006;7(12):1266–1273. 17110943 10.1038/ni1411

[55] Fahrenhold M, Rakic S, Classey J, et al TREM2 expression in the human brain: A marker of monocyte recruitment? Brain Pathol. 2018;28(5):595–602. 28987033 10.1111/bpa.12564 PMC6221091

[56] Liu W, Taso O, Wang R, et al Trem2 promotes anti-inflammatory responses in microglia and is suppressed under pro-inflammatory conditions. Hum Mol Genet. 2020;29(19):3224–3248. 32959884 10.1093/hmg/ddaa209 PMC7689298

[57] Nimmerjahn F, Gordan S, Lux A. Fcgammar dependent mechanisms of cytotoxic, agonistic, and neutralizing antibody activities. Trends Immunol. 2015;36(6):325–336. 25981969 10.1016/j.it.2015.04.005

[58] Yang W, Warrington NM, Taylor SJ, et al Sex differences in GBM revealed by analysis of patient imaging, transcriptome, and survival data. Sci Transl Med. 2019;11(473):eaao5253. 30602536 10.1126/scitranslmed.aao5253 PMC6502224

[59] Cancer Genome Atlas Research Network . Comprehensive genomic characterization defines human glioblastoma genes and core pathways. Nature. 2008;455(7216):1061–1068. 18772890 10.1038/nature07385 PMC2671642

[60] Lim M, Xia Y, Bettegowda C, Weller M. Current state of immunotherapy for glioblastoma. Nat Rev Clin Oncol. 2018;15(7):422–442. 29643471 10.1038/s41571-018-0003-5

[61] Bastola S, Pavlyukov MS, Yamashita D, et al Glioma-initiating cells at tumor edge gain signals from tumor core cells to promote their malignancy. Nat Commun. 2020;11(1):4660. 32938908 10.1038/s41467-020-18189-y PMC7494913

[62] Cullen SP, Henry CM, Kearney CJ, et al Fas/CD95-induced chemokines can serve as “find-me” signals for apoptotic cells. Mol Cell. 2013;49(6):1034–1048. 23434371 10.1016/j.molcel.2013.01.025

[63] Saavedra-Lopez E, Roig-Martinez M, Cribaro GP, et al Phagocytic glioblastoma-associated microglia and macrophages populate invading pseudopalisades. Brain Commun. 2020;2(1):fcz043. 32954312 10.1093/braincomms/fcz043 PMC7491442

[64] Huntington ND, Cursons J, Rautela J. The cancer-natural killer cell immunity cycle. Nat Rev Cancer. 2020;20(8):437–454. 32581320 10.1038/s41568-020-0272-z

[65] Thompson ED, Zahurak M, Murphy A, et al Patterns of PD-L1 expression and CD8 T cell infiltration in gastric adenocarcinomas and associated immune stroma. Gut. 2017;66(5):794–801. 26801886 10.1136/gutjnl-2015-310839 PMC4958028

[66] Gonzalez-Tablas Pimenta M, Otero A, Arandia Guzman DA, et al Tumor cell and immune cell profiles in primary human glioblastoma: Impact on patient outcome. Brain Pathol. 2021;31(2):365–380. 33314398 10.1111/bpa.12927 PMC8018082

[67] Landry AP, Balas M, Alli S, Spears J, Zador Z. Distinct regional ontogeny and activation of tumor associated macrophages in human glioblastoma. Sci Rep. 2020;10(1):19542. 33177572 10.1038/s41598-020-76657-3 PMC7658345

[68] Takenaka MC, Gabriely G, Rothhammer V, et al Control of tumor-associated macrophages and T cells in glioblastoma via AHR and CD39. Nat Neurosci. 2019;22(5):729–740. 30962630 10.1038/s41593-019-0370-y PMC8052632

[69] Mirzaei R, Yong VW. Microglia-T cell conversations in brain cancer progression. Trends Mol Med. 2022;28(11):951–963. 36075812 10.1016/j.molmed.2022.08.006

[70] Venkatesh HS, Morishita W, Geraghty AC, et al Electrical and synaptic integration of glioma into neural circuits. Nature. 2019;573(7775):539–545. 31534222 10.1038/s41586-019-1563-y PMC7038898

[71] Noorani I, Bradley A, de la Rosa J. CRISPR and transposon in vivo screens for cancer drivers and therapeutic targets. Genome Biol. 2020;21(1):204. 32811551 10.1186/s13059-020-02118-9 PMC7437018

[72] Noorani I . Genetically engineered mouse models of gliomas: Technological developments for translational discoveries. Cancers (Basel). 2019;11(9):1335. 31505839 10.3390/cancers11091335 PMC6770673

[73] Heynckes S, Daka K, Franco P, et al Crosslink between temozolomide and PD-L1 immune-checkpoint inhibition in glioblastoma multiforme. BMC Cancer. 2019;19(1):117. 30709339 10.1186/s12885-019-5308-y PMC6359796

[74] Wang Q, Hu B, Hu X, et al Tumor evolution of glioma-intrinsic gene expression subtypes associates with immunological changes in the microenvironment. Cancer Cell. 2017;32(1):42–56.e6. 28697342 10.1016/j.ccell.2017.06.003 PMC5599156

[75] Martinez-Lage M, Lynch TM, Bi Y, et al Immune landscapes associated with different glioblastoma molecular subtypes. Acta Neuropathol Commun. 2019;7(1):203. 31815646 10.1186/s40478-019-0803-6 PMC6902522

[76] Karschnia P, Young JS, Dono A, et al Prognostic validation of a new classification system for extent of resection in glioblastoma: A report of the RANO resect group. Neuro Oncol. 2023;25(5):940–954. 35961053 10.1093/neuonc/noac193 PMC10158281

