# Supplementary material for: Clinical impact of anti-inflammatory microglia and macrophage phenotypes at glioblastoma margins
Source: Brain Commun. 2023 Jun 2;5(3):fcad176. doi: 10.1093/braincomms/fcad176 (PMC10265726; doi:10.1093/braincomms/fcad176)
Supplement: fcad176_Supplementary_Data [file fcad176_supplementary_data.zip › Supplementary_table_legends.docx]

**Supplementary Table 1:** Clinical demographics of all patients included in this study.

**Supplementary Table 2:** List of antibodies used in this study.

**Supplementary Table 3:** Comparison of protein load (%) for each immune marker between regions of glioblastomas.

**Supplementary Table 4:** Correlations between markers across regions of glioblastomas. Spearman’s rank correlation was used for the analyses.
