# Supplementary material for: Clinical impact of anti-inflammatory microglia and macrophage phenotypes at glioblastoma margins
Source: Brain Commun. 2023 Jun 2;5(3):fcad176. doi: 10.1093/braincomms/fcad176 (PMC10265726; doi:10.1093/braincomms/fcad176)
Supplement: fcad176_Supplementary_Data [file fcad176_supplementary_data.zip › Suppl Table 2 - antibodies.docx]

**Supplementary Table 2 – Characteristics of the primary antibodies, immunohistochemistry conditions and expression of the staining**

| **Antigen**  **(human)** | **Company** | **Host** | **Pre-treatment** | **Dilution** | **Predominant neuropathological features / cell type stained** | **Immune functions** |
| --- | --- | --- | --- | --- | --- | --- |
| **Iba1** | Wako | Rabbit | Citrate buffer | 1/1000 | Microglia, perivascular macrophages | Cytoplasmic protein involved in cytoskeletal reorganization, membrane ruffling of the microglial processes and actin cross-linking needed for cell migration, reflecting microglial motility and migration properties. This marker is associated with physiological microglia^1^. High Iba1 expression was related to shorter survival times and faster tumour progression^2^. |
| **CD68** | Abcam | Mouse  Clone PG-M1 | 0.5% pronase | 1/50 | Microglia, perivascular macrophages | CD68 labels lysosomal and endosomal transmembrane glycoprotein of microglia, indicating phagocytic activity^3^ Its expression is associated with the proliferation rate of the tumour and faster tumour progression^2^ In vitro studies have demonstrated that gliomas actively recruit microglia^4^ and that their phagocytic activity may facilitate invasion and growth of the tumour^5^. |
| **HLA-DP,DQ,DR** | Dako | Mouse  Clone CR3/43 | Citrate buffer | 1/200 | Microglia, perivascular macrophages | HLA-DR is a Major Histocompatibility Class (MHC) II cell surface receptor which presents antigens to cells of the immune system eliciting an immune response, involved in the non-self recognition and upregulated in inflammation^6^. Its downregulation was described in experimental models ^7,8^ and in high-grade astrocytomas^9^, in response to anti-inflammatory cytokines^10,11^. |
| **CD64 (FcgRI)** | R&D Systems | Goat | EDTA buffer | 1/100 | Microglia, perivascular macrophages | Fcγ receptors are central effectors of immunoglobulins (IgG) mediated immune response^12^.  CD64 has high affinity for the Fc portion of IgG, triggering a monocyte/macrophage response^13^. CD64 is usually expressed with HLA-DR and associated with infiltrating macrophages^14^ activated by interferon-gamma (IFN-γ) to develop a tumour killing phenotype^15^.  CD32a and CD16 are low/medium affinity receptors for immune complex^12^. CD32a is the main activator of pro-inflammatory cytokine production and infiltration of CD16+ myeloid cells is associated with improved survival in patients with colorectal carcinoma^16^. |
| **CD32a (FcgRIIa)** | Abcam | Mouse  clone 13D7 | EDTA buffer | 1/2000 | Microglia, perivascular macrophages and in some neurons |  |
| **CD16 (FcgRIII)** | R&D Systems | Goat | EDTA buffer | 1/150 | Microglia, perivascular macrophages, some monocytes and neurons |  |
| **P2Y12** | Sigma Aldrich | Rabbit | Citrate buffer | 1:500 | Microglia | P2Y12 is a purinergic receptor specific to physiological microglia^1^. Its expression and localisation correspond to tumour grade and predominant stage of M1/M2 immune response in gliomas^17^. |
| **TREM2** | Sigma Aldrich | Rabbit | EDTA buffer | 1:100 | Monocytes, recruited macrophages | The role of the triggering receptor expressed on myeloid cells (TREM) 2 remains elusive in the human brain. Its expression has been identified on monocytes and recruited macrophages, acting as the gateway to phagocytosis^18^. |
| **CD163** | Serotec | Mouse  Clone EDHu-1 | EDTA buffer | 1:100 | Perivascular macrophages | CD163 is the haptoglobin-haemoglobin receptor specifically expressed by perivascular macrophages^19^ is upregulated in infiltrative tumour, suggesting CD163 as a marker of recruited macrophages^20^. |
| **CD206** | Abcam | Mouse  Clone 5C11 | EDTA buffer | 1:4000 | Perivascular macrophages | CD206 or mannose receptor is present on the surface of the tumour associated-macrophages and a potential biomarker for predicting prognosis in the context of hepatocellular carcinomas^21^. In the brain, its expression is associated with anti-inflammatory profile and specific for perivascular macrophages^22^ and thus might identify recruited macrophages. |
| **CD4** | Dako | Mouse  Clone 4B12 | Target Retrieval Solution | Ready-to-use | CD4^+^ T lymphocytes | CD4^+^ or helper T lymphocytes play an important role in modulating the immune responses to pathogens via MHC class II components. Their role in antitumour immunity remains controversial as they can suppress or promote the anti-tumour CTL response^23^. |
| **CD8** | Dako | Mouse  Clone C8/144B | Target Retrieval Solution | Ready-to-use | CD8^+^ T lymphocytes |  |
| **NKp46 (CD335)** | Invitrogen | Rabbit | EDTA buffer | 1:5000 | NK cells | CD335 or NCR1 targets the cytotoxicity-activating receptor that may contribute to the increased efficiency of activated natural killer (NK) cells to mediate tumour cell lysis. It has been associated with overall survival in solid cancers^24^. |
| **HIF1α** | Abcam | Mouse  Clone ESEE122 | EDTA buffer | 1:5000 | Ischemic cells | A master transcriptional regulator of the adaptive response to hypoxia, playing a key role in tumour angiogenesis^25^. |
| **PDL1** | Dako | Mouse  Clone 22C3 | Target Retrieval Solution | Ready-to-use | Tumour cells | PDL1 binds to the PD-1 receptor expressed on T cells, inhibiting T cell proliferation. Up-regulation of PD-1 ligands occurs in some tumours and signalling through this pathway can contribute to inhibition of active T-cell immune surveillance of tumours. |

References

1. Franco-Bocanegra DK, McAuley C, Nicoll JAR, Boche D. Molecular Mechanisms of Microglial Motility: Changes in Ageing and Alzheimer's Disease. *Cells.* 2019;8(6).

2. Noorani I, Petty G, Grundy PL, et al. Novel association between microglia and stem cells in human gliomas: A contributor to tumour proliferation? *J Pathol Clin Res.* 2015;1(2):67-75.

3. Rabinowitz SS, Gordon S. Macrosialin, a macrophage-restricted membrane sialoprotein differentially glycosylated in response to inflammatory stimuli. *J Exp Med.* 1991;174(4):827-836.

4. Okada M, Saio M, Kito Y, et al. Tumor-associated macrophage/microglia infiltration in human gliomas is correlated with MCP-3, but not MCP-1. *Int J Oncol.* 2009;34(6):1621-1627.

5. Ghosh A, Chaudhuri S. Microglial action in glioma: a boon turns bane. *Immunol Lett.* 2010;131(1):3-9.

6. Styren SD, Civin WH, Rogers J. Molecular, cellular, and pathologic characterization of HLA-DR immunoreactivity in normal elderly and Alzheimer's disease brain. *Exp Neurol.* 1990;110(1):93-104.

7. Badie B, Bartley B, Schartner J. Differential expression of MHC class II and B7 costimulatory molecules by microglia in rodent gliomas. *J Neuroimmunol.* 2002;133(1-2):39-45.

8. Billingham C, Powell MR, Jenner KA, et al. Rat astrocytic tumour cells are associated with an anti-inflammatory microglial phenotype in an organotypic model. *Neuropathol Appl Neurobiol.* 2013;39(3):243-255.

9. Graeber MB, Scheithauer BW, Kreutzberg GW. Microglia in brain tumors. *Glia.* 2002;40(2):252-259.

10. Platten M, Wick W, Weller M. Malignant glioma biology: role for TGF-beta in growth, motility, angiogenesis, and immune escape. *Microsc Res Tech.* 2001;52(4):401-410.

11. Taniguchi Y, Ono K, Yoshida S, Tanaka R. Antigen-presenting capability of glial cells under glioma-harboring conditions and the effect of glioma-derived factors on antigen presentation. *J Neuroimmunol.* 2000;111(1-2):177-185.

12. Nimmerjahn F, Gordan S, Lux A. FcgammaR dependent mechanisms of cytotoxic, agonistic, and neutralizing antibody activities. *Trends Immunol.* 2015;36(6):325-336.

13. Vogelpoel LT, Baeten DL, de Jong EC, den Dunnen J. Control of cytokine production by human fc gamma receptors: implications for pathogen defense and autoimmunity. *Front Immunol.* 2015;6:79.

14. Wallace PK, Romet-Lemonne JL, Chokri M, Kasper LH, Fanger MW, Fadul CE. Production of macrophage-activated killer cells for targeting of glioblastoma cells with bispecific antibody to FcgammaRI and the epidermal growth factor receptor. *Cancer Immunol Immunother.* 2000;49(9):493-503.

15. Ritchie D, Mileshkin L, Wall D, et al. In vivo tracking of macrophage activated killer cells to sites of metastatic ovarian carcinoma. *Cancer Immunol Immunother.* 2007;56(2):155-163.

16. Sconocchia G, Zlobec I, Lugli A, et al. Tumor infiltration by FcgammaRIII (CD16)+ myeloid cells is associated with improved survival in patients with colorectal carcinoma. *Int J Cancer.* 2011;128(11):2663-2672.

17. Zhu C, Kros JM, van der Weiden M, Zheng P, Cheng C, Mustafa DA. Expression site of P2RY12 in residential microglial cells in astrocytomas correlates with M1 and M2 marker expression and tumor grade. *Acta Neuropathol Commun.* 2017;5(1):4.

18. Fahrenhold M, Rakic S, Classey J, et al. TREM2 expression in the human brain: a marker of monocyte recruitment? *Brain Pathol.* 2018;28(5):595-602.

19. Kristiansen M, Graversen JH, Jacobsen C, et al. Identification of the haemoglobin scavenger receptor. *Nature.* 2001;409(6817):198-201.

20. Lu-Emerson C, Snuderl M, Kirkpatrick ND, et al. Increase in tumor-associated macrophages after antiangiogenic therapy is associated with poor survival among patients with recurrent glioblastoma. *Neuro Oncol.* 2013;15(8):1079-1087.

21. Zhu F, Li X, Jiang Y, et al. GdCl3 suppresses the malignant potential of hepatocellular carcinoma by inhibiting the expression of CD206 in tumor‑associated macrophages. *Oncol Rep.* 2015;34(5):2643-2655.

22. Galea I, Palin K, Newman TA, Van Rooijen N, Perry VH, Boche D. Mannose receptor expression specifically reveals perivascular macrophages in normal, injured, and diseased mouse brain. *Glia.* 2005;49(3):375-384.

23. Ahrends T, Borst J. The opposing roles of CD4(+) T cells in anti-tumour immunity. *Immunology.* 2018;154(4):582-592.

24. Nersesian S, Schwartz SL, Grantham SR, et al. NK cell infiltration is associated with improved overall survival in solid cancers: A systematic review and meta-analysis. *Transl Oncol.* 2021;14(1):100930.

25. Kaur B, Khwaja FW, Severson EA, Matheny SL, Brat DJ, Van Meir EG. Hypoxia and the hypoxia-inducible-factor pathway in glioma growth and angiogenesis. *Neuro Oncol.* 2005;7(2):134-153.
