# Supplementary material for: Clinical impact of anti-inflammatory microglia and macrophage phenotypes at glioblastoma margins
Source: Brain Commun. 2023 Jun 2;5(3):fcad176. doi: 10.1093/braincomms/fcad176 (PMC10265726; doi:10.1093/braincomms/fcad176)
Supplement: fcad176_Supplementary_Data [file fcad176_supplementary_data.zip › Supplementary table 3, protein loads across regions.docx]

Supplementary Table 3 . Protein load (%) for each marker assessed in the three areas.

| Marker | Core | Infiltrating zone | Leading edge | *P* value^1^ | *P* value^2^ | *P* value^3^ |
| --- | --- | --- | --- | --- | --- | --- |
| Iba1 | 6.110 ± 3.257 | 5.078 ± 3.292 | 2.410 ± 2.317 | 0.265 | **<0.001** | **<0.001** |
| CD68 | 1.365 ± 0.978 | 1.020 ± 0.964 | 0.682 ± 0.800 | 0.105 | **<0.001** | **0.003** |
| HLA-DR | 1.295 ± 4.565 | 0.265 ± 0.708 | 0.077 ± 0.290 | 0.090 | **<0.001** | **0.002** |
| CD64* | 3.025 ± 3.028 | 2.750 ± 3.522 | 1.958 ± 1.658 | ns | ns | ns |
| CD32a | 7.411 ± 4.638 | 6.506 ± 3.778 | 3.764 ± 4.336 | 1.000 | **<0.001** | **<0.001** |
| CD16 | 4.926 ± 4.421 | 3.947 ± 4.691 | 1.306 ± 1.784 | 0.296 | **<0.001** | **<0.001** |
| P2RY12 | 0.588 ± 0.663 | 0.837 ± 0.907 | 0.666 ± 0.398 | **0.037** | **0.037** | **1.000** |
| TREM2 | 0.227 ± 0.317 | 0.148 ± 0.151 | 0.094 ± 0.220 | 0.277 | **<0.001** | **0.001** |
| CD163 | 3.089 ± 3.085 | 1.519 ± 1.699 | 0.591 ± 1.036 | **0.002** | **<0.001** | **<0.001** |
| CD206 | 1.853 ± 3.021 | 1.473 ± 2.163 | 1.026 ± 1.451 | 0.772 | **0.008** | 0.206 |
| CD4 | 0.420 ± 0.604 | 0.356 ± 0.810 | 0.230 ± 1.198 | 0.060 | **<0.001** | **0.012** |
| CD8* | 0.730 ± 0.944 | 0.520 ± 0.576 | 0.056 ± 0.889 | ns | ns | ns |
| CD335 | 0.468 ± 0.297 | 0.422 ± 0.417 | 0.394 ± 0.496 | 0.298 | **0.003** | 0.324 |
| HIF1α | 3.028 ± 2.498 | 2.131 ± 1.742 | 1.319 ± 1.081 | 0.182 | **<0.001** | **0.029** |
| PDL1 (22C3) | 0.004 ± 0.005 | 0.002 ± 0.005 | 0.001 ± 0.003 | **0.037** | **<0.001** | 0.413 |

Values are presented as mean ± SD, *P* value by Kruskal-Wallis test with pairwise comparison adjusted by the Bonferroni correction for multiple tests

Significant P value in bold.

^1^core *vs*. infiltrating zone, ^2^core *vs*. leading edge, ^3^infiltrating zone *vs*. leading edge

*non-significant Kruskal-Wallis test: CD64, *P* = 0.087; CD8, *P* = 0.526
