# Supplementary material for: Clinical impact of anti-inflammatory microglia and macrophage phenotypes at glioblastoma margins
Source: Brain Commun. 2023 Jun 2;5(3):fcad176. doi: 10.1093/braincomms/fcad176 (PMC10265726; doi:10.1093/braincomms/fcad176)
Supplement: fcad176_Supplementary_Data [file fcad176_supplementary_data.zip › Supplementary Table 4, correlations.docx]

|  | **Core** | **Infiltrating Margin** | **Leading Edge** |
| --- | --- | --- | --- |
| Iba1 vs CD68 | \| ρ= 0.351** \| \| --- \| \| P= 0.007 \| | \| ρ= 0.545*** \| \| --- \| \| P<.001 \| | \| ρ=0.396** \| \| --- \| \| P=0.002 \| |
| Iba1 vs HLA-DR | \| ρ=0.385** \| \| --- \| \| P=0.004 \| | \| ρ=0.253 \| \| --- \| \| P=0.065 \| | \| ρ=0.269 \| \| --- \| \| P=0.051 \| |
| Iba1 vs CD64 | \| ρ=0.281* \| \| --- \| \| P=0.036 \| | \| ρ=0.089 \| \| --- \| \| P=0.528 \| | \| ρ=-0.058 \| \| --- \| \| P=0.676 \| |
| Iba1 vs CD32a | \| ρ=.670** \| \| --- \| \| P<.001 \| | \| ρ=0.792*** \| \| --- \| \| P<.001 \| | \| ρ=0.582*** \| \| --- \| \| P<.001 \| |
| Iba1 vs CD16 | \| ρ=0.606*** \| \| --- \| \| P<.001 \| | \| ρ=0.478*** \| \| --- \| \| P<.001 \| | \| ρ=0.527*** \| \| --- \| \| P<.001 \| |
| Iba1 vs P2YR12 | \| ρ=0.489*** \| \| --- \| \| P<.001 \| | \| ρ=0.003 \| \| --- \| \| P=0.984 \| | \| ρ=0.215 \| \| --- \| \| P=0.105 \| |
| Iba1 vs CD163 | \| ρ=0.411** \| \| --- \| \| P=0.001 \| | \| ρ=0.564*** \| \| --- \| \| P<.001 \| | \| ρ=0.189 \| \| --- \| \| P=0.155 \| |
| Iba1 vs CD206 | \| ρ=0.126 \| \| --- \| \| P=0.346 \| | \| ρ=0.183 \| \| --- \| \| P=0.186 \| | \| ρ=0.068 \| \| --- \| \| P=0.614 \| |
| Iba1 vs TREM2 | \| ρ=0.03 \| \| --- \| \| P=0.826 \| | \| ρ=0.308* \| \| --- \| \| P=0.022 \| | \| ρ=0.392** \| \| --- \| \| P=0.002 \| |
| Iba1 vs CD335 | \| ρ=-0.334* \| \| --- \| \| P=0.011 \| | \| ρ=-0.109 \| \| --- \| \| P=0.428 \| | \| ρ=0.063 \| \| --- \| \| P=0.638 \| |
| Iba1 vs CD4 | \| ρ=0.628*** \| \| --- \| \| P<.001 \| | \| ρ=0.676*** \| \| --- \| \| P<.001 \| | \| ρ=0.763*** \| \| --- \| \| P<.001 \| |
| Iba1 vs CD8 | \| ρ=0.239 \| \| --- \| \| P=0.076 \| | \| ρ=0.563*** \| \| --- \| \| P<.001 \| | \| ρ=0.091 \| \| --- \| \| P=0.505 \| |
| Iba1 vs HIF-1a | \| ρ=0.320* \| \| --- \| \| P=0.014 \| | \| ρ=0.320* \| \| --- \| \| P=0.017 \| | \| ρ=0.557*** \| \| --- \| \| P<.001 \| |
| Iba1 vs PD-L1 | \| ρ=-0.295* \| \| --- \| \| P=0.026 \| | \| ρ=-0.309* \| \| --- \| \| P=0.022 \| | \| ρ=0.093 \| \| --- \| \| P=0.489 \| |
| CD68 *vs* HLA-DR | \| ρ=0.371** \| \| --- \| \| P=0.005 \| | \| ρ=0.552*** \| \| --- \| \| P<.001 \| | \| ρ=0.484*** \| \| --- \| \| P<.001 \| |
| CD68 *vs* CD64 | \| ρ=0.028 \| \| --- \| \| P=0.835 \| | \| ρ=0.108 \| \| --- \| \| P=0.444 \| | \| ρ=0.276* \| \| --- \| \| P=0.042 \| |
| CD68 *vs* CD32a | \| ρ=0.383** \| \| --- \| \| P=0.003 \| | \| ρ=0.605*** \| \| --- \| \| P<.001 \| | \| ρ=0.572*** \| \| --- \| \| P<.001 \| |
| CD68 *vs* CD16 | \| ρ=0.184 \| \| --- \| \| P=0.17 \| | \| ρ=0.485*** \| \| --- \| \| P<.001 \| | \| ρ=0.426*** \| \| --- \| \| P<.001 \| |
| CD68 *vs* P2RY12 | \| ρ=0.145 \| \| --- \| \| P=0.278 \| | \| ρ=-0.023 \| \| --- \| \| P=0.869 \| | \| ρ=0.092 \| \| --- \| \| P=0.489 \| |
| CD68 *vs* CD163 | \| ρ=0.320* \| \| --- \| \| P=0.014 \| | \| ρ=0.453*** \| \| --- \| \| P<.001 \| | \| ρ=0.592*** \| \| --- \| \| P<.001 \| |
| CD68 *vs* CD206 | \| ρ=0.089 \| \| --- \| \| P=0.504 \| | \| ρ=0.07 \| \| --- \| \| P=0.616 \| | \| ρ=0.285* \| \| --- \| \| P=0.029 \| |
| CD68 *vs* TREM2 | \| ρ=0.087 \| \| --- \| \| P=0.518 \| | \| ρ=0.314* \| \| --- \| \| P=0.02 \| | \| ρ=0.596*** \| \| --- \| \| P<.001 \| |
| CD68 *vs* CD335 | \| ρ=0.112 \| \| --- \| \| P=0.409 \| | \| ρ=0.196 \| \| --- \| \| P=0.152 \| | \| ρ=0.353** \| \| --- \| \| P=0.006 \| |
| CD68 *vs* CD4 | \| ρ=0.429*** \| \| --- \| \| P<.001 \| | \| ρ=0.593*** \| \| --- \| \| P<.001 \| | \| ρ=0.526*** \| \| --- \| \| P<.001 \| |
| CD68 *vs* CD8 | \| ρ=0.228 \| \| --- \| \| P=0.091 \| | \| ρ=0.496*** \| \| --- \| \| P<.001 \| | \| ρ=0.511*** \| \| --- \| \| P<.001 \| |
| CD68 *vs* HIF-1a | \| ρ=0.339** \| \| --- \| \| P=0.009 \| | \| ρ=0.404** \| \| --- \| \| P=0.002 \| | \| ρ=0.234 \| \| --- \| \| P=0.075 \| |
| CD68 *vs* PD-L1 | \| ρ=0.077 \| \| --- \| \| P=0.57 \| | \| ρ=-0.048 \| \| --- \| \| P=0.725 \| | \| ρ=0.401** \| \| --- \| \| P=0.002 \| |
| HLA-DR *vs* CD64 | \| ρ=0.242 \| \| --- \| \| P=0.078 \| | \| ρ=0.137 \| \| --- \| \| P=0.331 \| | \| ρ=0.167 \| \| --- \| \| P=0.245 \| |
| HLA-DR *vs* CD32a | \| ρ=0.471*** \| \| --- \| \| P<.001 \| | \| ρ=0.331* \| \| --- \| \| P=0.014 \| | \| ρ=0.414** \| \| --- \| \| P=0.002 \| |
| HLA-DR *vs* CD16 | \| ρ=0.286* \| \| --- \| \| P=0.036 \| | \| ρ=0.106 \| \| --- \| \| P=0.454 \| | \| ρ=0.339* \| \| --- \| \| P=0.013 \| |
| HLA-DR *vs* P2RY12 | \| ρ=0.016 \| \| --- \| \| P=0.906 \| | \| ρ=-0.019 \| \| --- \| \| P=0.89 \| | \| ρ=0.26 \| \| --- \| \| P=0.057 \| |
| HLA-DR *vs* CD163 | \| ρ=0.544*** \| \| --- \| \| P<.001 \| | \| ρ=0.212 \| \| --- \| \| P=0.128 \| | \| ρ=0.346* \| \| --- \| \| P=0.01 \| |
| HLA-DR *vs* CD206 | \| ρ=0.006 \| \| --- \| \| P=0.963 \| | \| ρ=-0.047 \| \| --- \| \| P=0.739 \| | \| ρ=0.009 \| \| --- \| \| P=0.948 \| |
| HLA-DR *vs* TREM2 | \| ρ=0.039 \| \| --- \| \| P=0.776 \| | \| ρ=0.206 \| \| --- \| \| P=0.131 \| | \| ρ=0.385** \| \| --- \| \| P=0.004 \| |
| HLA-DR *vs* CD335 | \| ρ=-0.073 \| \| --- \| \| P=0.601 \| | \| ρ=0.226 \| \| --- \| \| P=0.1 \| | \| ρ=0.286* \| \| --- \| \| P=0.036 \| |
| HLA-DR *vs* CD4 | \| ρ=0.256 \| \| --- \| \| P=0.061 \| | \| ρ=0.212 \| \| --- \| \| P=0.12 \| | \| ρ=0.340* \| \| --- \| \| P=0.014 \| |
| HLA-DR *vs* CD8 | \| ρ=0.174 \| \| --- \| \| P=0.213 \| | \| ρ=0.477*** \| \| --- \| \| P<.001 \| | \| ρ=0.340* \| \| --- \| \| P=0.014 \| |
| HLA-DR *vs* HIF-1a | \| ρ=-0.011 \| \| --- \| \| P=0.934 \| | \| ρ=0.113 \| \| --- \| \| P=0.415 \| | \| ρ=0.239 \| \| --- \| \| P=0.082 \| |
| HLA-DR *vs* PD-L1 | \| ρ=0.091 \| \| --- \| \| P=0.514 \| | \| ρ=0.212 \| \| --- \| \| P=0.124 \| | \| ρ=0.387** \| \| --- \| \| P=0.004 \| |
| CD64 *vs* CD32a | \| ρ=0.162 \| \| --- \| \| P=0.233 \| | \| ρ=0.024 \| \| --- \| \| P=0.863 \| | \| ρ=0.314* \| \| --- \| \| P=0.019 \| |
| CD64 *vs* CD16 | \| ρ=0.392** \| \| --- \| \| P=0.003 \| | \| ρ=0.367** \| \| --- \| \| P=0.007 \| | \| ρ=0.262 \| \| --- \| \| P=0.056 \| |
| CD64 *vs* P2RY12 | \| ρ=-0.04 \| \| --- \| \| P=0.769 \| | \| ρ=-0.109 \| \| --- \| \| P=0.435 \| | \| ρ=0.157 \| \| --- \| \| P=0.252 \| |
| CD64 *vs* CD163 | \| ρ=0.272* \| \| --- \| \| P=0.043 \| | \| ρ=0.044 \| \| --- \| \| P=0.761 \| | \| ρ=0.315* \| \| --- \| \| P=0.019 \| |
| CD64 *vs* CD206 | \| ρ=0.135 \| \| --- \| \| P=0.321 \| | \| ρ=0.295* \| \| --- \| \| P=0.036 \| | \| ρ=0.245 \| \| --- \| \| P=0.072 \| |
| CD64 *vs* TREM2 | \| ρ=0.068 \| \| --- \| \| P=0.616 \| | \| ρ=-0.02 \| \| --- \| \| P=0.886 \| | \| ρ=0.282* \| \| --- \| \| P=0.037 \| |
| CD64 *vs* CD335 | \| ρ=-0.254 \| \| --- \| \| P=0.061 \| | \| ρ=-0.09 \| \| --- \| \| P=0.517 \| | \| ρ=0.189 \| \| --- \| \| P=0.166 \| |
| CD64 *vs* CD4 | \| ρ=0.037 \| \| --- \| \| P=0.79 \| | \| ρ=0.03 \| \| --- \| \| P=0.829 \| | \| ρ=0.09 \| \| --- \| \| P=0.519 \| |
| CD64 *vs* CD8 | \| ρ=-0.016 \| \| --- \| \| P=0.91 \| | \| ρ=0.137 \| \| --- \| \| P=0.324 \| | \| ρ=0.263 \| \| --- \| \| P=0.057 \| |
| CD64 *vs* HIF-1a | \| ρ=-0.179 \| \| --- \| \| P= 0.186 \| | \| ρ=-0.203 \| \| --- \| \| P=0.142 \| | \| ρ=-0.097 \| \| --- \| \| P=0.479 \| |
| CD64 *vs* PD-L1 | \| ρ=-0.008 \| \| --- \| \| P=0.952 \| | \| ρ=0.077 \| \| --- \| \| P=0.583 \| | \| ρ=0.267 \| \| --- \| \| P=0.051 \| |
| CD32a *vs* CD16 | \| ρ=0.670*** \| \| --- \| \| P<.001 \| | \| ρ=0.480*** \| \| --- \| \| P<.001 \| | \| ρ=0.405** \| \| --- \| \| P=0.002 \| |
| CD32a *vs* P2RY12 | \| ρ=0.406** \| \| --- \| \| P=0.001 \| | \| ρ=0.067 \| \| --- \| \| P=0.626 \| | \| ρ=0.157 \| \| --- \| \| P=0.235 \| |
| CD32a *vs* CD163 | \| ρ=.451*** \| \| --- \| \| P<.001 \| | \| ρ=0.517*** \| \| --- \| \| P<.001 \| | \| ρ=0.521*** \| \| --- \| \| P<.001 \| |
| CD32a *vs* CD206 | \| ρ=0.039 \| \| --- \| \| P=0.773 \| | \| ρ=0.008 \| \| --- \| \| P=0.954 \| | \| ρ=0.191 \| \| --- \| \| P=0.147 \| |
| CD32a *vs* TREM2 | \| ρ=0.083 \| \| --- \| \| P=0.534 \| | \| ρ=0.396** \| \| --- \| \| P=0.002 \| | \| ρ=0.632*** \| \| --- \| \| P<.001 \| |
| CD32a *vs* CD335 | \| ρ=-0.182 \| \| --- \| \| P=0.171 \| | \| ρ=-0.083 \| \| --- \| \| P=0.541 \| | \| ρ=0.271* \| \| --- \| \| P=0.038 \| |
| CD32a *vs* CD4 | \| ρ=0.631*** \| \| --- \| \| P=<.001 \| | \| ρ=0.651*** \| \| --- \| \| P<.001 \| | \| ρ=0.640*** \| \| --- \| \| P<.001 \| |
| CD32a *vs* CD8 | \| ρ=0.122 \| \| --- \| \| P=0.366 \| | \| ρ=0.511*** \| \| --- \| \| P<.001 \| | \| ρ=0.435*** \| \| --- \| \| P<.001 \| |
| CD32a *vs* HIF-1a | \| ρ=0.478*** \| \| --- \| \| P<.001 \| | \| ρ=0.575*** \| \| --- \| \| P<.001 \| | \| ρ=0.331* \| \| --- \| \| P=0.01 \| |
| CD32a *vs* PD-L1 | \| ρ=-0.081 \| \| --- \| \| P=0.547 \| | \| ρ=-0.217 \| \| --- \| \| P=0.109 \| | \| ρ=0.405** \| \| --- \| \| P=0.002 \| |
| CD16 *vs* P2RY12 | \| ρ=0.169 \| \| --- \| \| P=0.204 \| | \| ρ=0.006 \| \| --- \| \| P=0.963 \| | \| ρ=0.219 \| \| --- \| \| P=0.099 \| |
| CD16 *vs* CD163 | \| ρ=0.567*** \| \| --- \| \| P<.001 \| | \| ρ=0.356** \| \| --- \| \| P=0.01 \| | \| ρ=0.246 \| \| --- \| \| P=0.063 \| |
| CD16 *vs* CD206 | \| ρ=0.159 \| \| --- \| \| P=0.237 \| | \| ρ=0.197 \| \| --- \| \| P=0.162 \| | \| ρ=0.134 \| \| --- \| \| P=0.314 \| |
| CD16 *vs* TREM2 | \| ρ=-0.014 \| \| --- \| \| P=0.917 \| | \| ρ=0.235 \| \| --- \| \| P=0.087 \| | \| ρ=0.346** \| \| --- \| \| P=0.008 \| |
| CD16 *vs* CD335 | \| ρ=-0.306* \| \| --- \| \| P=0.021 \| | \| ρ=-0.23 \| \| --- \| \| P=0.09 \| | \| ρ=-0.093 \| \| --- \| \| P=0.486 \| |
| CD16 *vs* CD4 | \| ρ=0.537*** \| \| --- \| \| P<.001 \| | \| ρ=0.668*** \| \| --- \| \| P<.001 \| | \| ρ=0.599*** \| \| --- \| \| P<.001 \| |
| CD16 *vs* CD8 | \| ρ=0.161 \| \| --- \| \| P=0.235 \| | \| ρ=0.279* \| \| --- \| \| P=0.039 \| | \| ρ=-0.025 \| \| --- \| \| P=0.854 \| |
| CD16 *vs* HIF-1a | \| ρ=0.449*** \| \| --- \| \| P<.001 \| | \| ρ=0.425** \| \| --- \| \| P=0.001 \| | \| ρ=0.319* \| \| --- \| \| P=0.015 \| |
| CD16 *vs* PD-L1 | \| ρ=-0.360** \| \| --- \| \| P=0.006 \| | \| ρ=-0.199 \| \| --- \| \| P=0.149 \| | \| ρ=0.031 \| \| --- \| \| P=0.817 \| |
| P2RY12 *vs* CD163 | \| ρ=-0.167 \| \| --- \| \| P=0.211 \| | \| ρ=-0.209 \| \| --- \| \| P=0.129 \| | \| ρ=0.01 \| \| --- \| \| P=0.941 \| |
| P2RY12 *vs* CD206 | \| ρ=-0.142 \| \| --- \| \| P=0.286 \| | \| ρ=-0.031 \| \| --- \| \| P=0.826 \| | \| ρ=-0.033 \| \| --- \| \| P=0.806 \| |
| P2RY12 *vs* TREM2 | \| ρ=0.153 \| \| --- \| \| P=0.247 \| | \| ρ=0.027 \| \| --- \| \| P=0.845 \| | \| ρ=0.211 \| \| --- \| \| P=0.108 \| |
| P2RY12 *vs* CD335 | \| ρ=-0.029 \| \| --- \| \| P=0.831 \| | \| ρ=0.003 \| \| --- \| \| P=0.98 \| | \| ρ=0.205 \| \| --- \| \| P=0.119 \| |
| P2RY12 *vs* CD4 | \| ρ=0.286* \| \| --- \| \| P=0.029 \| | \| ρ=-0.087 \| \| --- \| \| P=0.521 \| | \| ρ=0.087 \| \| --- \| \| P=0.522 \| |
| P2RY12 *vs* CD8 | \| ρ=0.254 \| \| --- \| \| P=0.057 \| | \| ρ=-0.09 \| \| --- \| \| P=0.508 \| | \| ρ=0.115 \| \| --- \| \| P=0.395 \| |
| P2RY12 *vs* HIF-1a | \| ρ=0.369** \| \| --- \| \| P=0.004 \| | \| ρ=0.037 \| \| --- \| \| P=0.782 \| | \| ρ=0.17 \| \| --- \| \| P=0.198 \| |
| P2RY12 *vs* PD-L1 | \| ρ=-0.107 \| \| --- \| \| P=0.425 \| | \| ρ=0.02 \| \| --- \| \| P=0.885 \| | \| ρ=-0.091 \| \| --- \| \| P=0.496 \| |
| CD163 *vs* CD206 | \| ρ=0.433*** \| \| --- \| \| P<.001 \| | \| ρ=0.320* \| \| --- \| \| P=0.02 \| | \| ρ=0.375** \| \| --- \| \| P=0.003 \| |
| CD163 *vs* TREM2 | \| ρ=-0.083 \| \| --- \| \| P=0.536 \| | \| ρ=0.392** \| \| --- \| \| P=0.003 \| | \| ρ=0.557*** \| \| --- \| \| P<.001 \| |
| CD163 *vs* CD335 | \| ρ=-0.086 \| \| --- \| \| P=0.526 \| | \| ρ=0.121 \| \| --- \| \| P=0.382 \| | \| ρ=0.430*** \| \| --- \| \| P<.001 \| |
| CD163 *vs* CD4 | \| ρ=0.413** \| \| --- \| \| P=0.001 \| | \| ρ=0.538*** \| \| --- \| \| P<.001 \| | \| ρ=0.275* \| \| --- \| \| P=0.039 \| |
| CD163 *vs* CD8 | \| ρ=0.371** \| \| --- \| \| P=0.005 \| | \| ρ=0.665*** \| \| --- \| \| P<.001 \| | \| ρ=0.528*** \| \| --- \| \| P<.001 \| |
| CD163 *vs* HIF-1a | \| ρ=0.086 \| \| --- \| \| P=0.523 \| | \| ρ=0.136 \| \| --- \| \| P=0.328 \| | \| ρ=0.016 \| \| --- \| \| P=0.903 \| |
| CD163 *vs* PD-L1 | \| ρ=-0.142 \| \| --- \| \| P=0.29 \| | \| ρ=0.143 \| \| --- \| \| P=0.301 \| | \| ρ=0.344* \| \| --- \| \| P=0.008 \| |
| CD206 *vs* TREM2 | \| ρ=-0.239 \| \| --- \| \| P=0.07 \| | \| ρ=-0.117 \| \| --- \| \| P=0.398 \| | \| ρ=0.248 \| \| --- \| \| P=0.058 \| |
| CD206 *vs* CD335 | \| ρ=0.138 \| \| --- \| \| P=0.305 \| | \| ρ=0.003 \| \| --- \| \| P=0.985 \| | \| ρ=-0.008 \| \| --- \| \| P=0.953 \| |
| CD206 *vs* CD4 | \| ρ=0.227 \| \| --- \| \| P=0.089 \| | \| ρ=0.288* \| \| --- \| \| P=0.035 \| | \| ρ=0.26 \| \| --- \| \| P=0.05 \| |
| CD206 *vs* CD8 | \| ρ=0.399** \| \| --- \| \| P=0.002 \| | \| ρ=0.214 \| \| --- \| \| P=0.121 \| | \| ρ=-0.051 \| \| --- \| \| P=0.706 \| |
| CD206 *vs* HIF-1a | \| ρ=-0.208 \| \| --- \| \| P=0.118 \| | \| ρ=-0.302* \| \| --- \| \| P=0.027 \| | \| ρ=-0.321* \| \| --- \| \| P=0.013 \| |
| CD206 *vs* PD-L1 | \| ρ=-0.196 \| \| --- \| \| P=0.143 \| | \| ρ=-0.058 \| \| --- \| \| P=0.679 \| | \| ρ=0.013 \| \| --- \| \| P=0.921 \| |
| TREM2 *vs* CD335 | \| ρ=0.168 \| \| --- \| \| P=0.208 \| | \| ρ=0.237 \| \| --- \| \| P=0.078 \| | \| ρ=0.484*** \| \| --- \| \| P<.001 \| |
| TREM2 *vs* CD4 | \| ρ=0.016 \| \| --- \| \| P=0.904 \| | \| ρ=0.417** \| \| --- \| \| P=0.001 \| | \| ρ=0.503*** \| \| --- \| \| P<.001 \| |
| TREM2 *vs* CD8 | \| ρ=-0.297* \| \| --- \| \| P=0.025 \| | \| ρ=0.405** \| \| --- \| \| P=0.002 \| | \| ρ=0.376** \| \| --- \| \| P=0.004 \| |
| TREM2 *vs* HIF-1a | \| ρ=0.333** \| \| --- \| \| P=0.01 \| | \| ρ=0.557*** \| \| --- \| \| P<.001 \| | \| ρ=0.412** \| \| --- \| \| P=0.001 \| |
| TREM2 *vs* PD-L1 | \| ρ=0.224 \| \| --- \| \| P=0.09 \| | \| ρ=-0.026 \| \| --- \| \| P=0.848 \| | \| ρ=0.436*** \| \| --- \| \| P<.001 \| |
| CD335 *vs* CD4 | \| ρ=-0.076 \| \| --- \| \| P=0.575 \| | \| ρ=-0.03 \| \| --- \| \| P=0.823 \| | \| ρ=0.143 \| \| --- \| \| P=0.288 \| |
| CD335 *vs* CD8 | \| ρ=0.003 \| \| --- \| \| P=0.982 \| | \| ρ=0.136 \| \| --- \| \| P=0.314 \| | \| ρ=0.485*** \| \| --- \| \| P<.001 \| |
| CD335 *vs* HIF-1a | \| ρ=0.117 \| \| --- \| \| P=0.384 \| | \| ρ=-0.131 \| \| --- \| \| P=0.331 \| | \| ρ=0.256 \| \| --- \| \| P=0.051 \| |
| CD335 *vs* PD-L1 | \| ρ=0.205 \| \| --- \| \| P=0.125 \| | \| ρ=0.393** \| \| --- \| \| P=0.003 \| | \| ρ=0.545*** \| \| --- \| \| P<.001 \| |
| CD4 *vs* CD8 | \| ρ=0.215 \| \| --- \| \| P=0.111 \| | \| ρ=0.535*** \| \| --- \| \| P=<.001 \| | \| ρ=0.18 \| \| --- \| \| P=0.188 \| |
| CD4 *vs* HIF-1a | \| ρ=0.454*** \| \| --- \| \| P<.001 \| | \| ρ=.519*** \| \| --- \| \| P<.001 \| | \| ρ=0.499*** \| \| --- \| \| P<.001 \| |
| CD4 *vs* PD-L1 | \| ρ=-0.325* \| \| --- \| \| P=0.014 \| | \| ρ=-0.249 \| \| --- \| \| P=0.064 \| | \| ρ=0.255 \| \| --- \| \| P=0.058 \| |
| CD8 *vs* HIF-1a | \| ρ=-0.01 \| \| --- \| \| P=0.939 \| | \| ρ=0.191 \| \| --- \| \| P=0.154 \| | \| ρ=0.17 \| \| --- \| \| P=0.205 \| |
| CD8 *vs* PD-L1 | \| ρ=-0.183 \| \| --- \| \| P=0.177 \| | \| ρ=0.138 \| \| --- \| \| P=0.311 \| | \| ρ=0.490*** \| \| --- \| \| P<.001 \| |
| HIF-1a *vs* PD-L1 | \| ρ=-0.134 \| \| --- \| \| P=0.316 \| | \| ρ=-0.237 \| \| --- \| \| P=0.079 \| | \| ρ=0.172 \| \| --- \| \| P=0.197 \| |
